# Evaluating the Management of chronic Pelvic girdle Pain following pregnancy (EMaPP): study protocol for a randomised controlled feasibility trial to compare a customised pelvic orthosis with standard care

**DOI:** 10.1136/bmjopen-2022-063767

**Published:** 2022-08-04

**Authors:** Bradley John Halliday, Sarah Chatfield, Lee Cameron, Joanne Hosking, Jill Shawe, Annie Hawton, Christopher Hayward, Kirsty Carter, Jennifer A Freeman

**Affiliations:** 1 Faculty of Health, University of Plymouth, Plymouth, UK; 2 Spinal Trauma & Orthopaedics, Aneurin Bevan Health Board, Newport, UK; 3 Peninsula Clinical Trials Unit, University of Plymouth, Plymouth, UK; 4 Clinical School, Royal Cornwall Hospitals NHS Trust, Truro, UK; 5 Health Economics Group, University of Exeter, Exeter, UK; 6 Cornwall Partnership NHS Foundation Trust, Bodmin, UK

**Keywords:** REHABILITATION MEDICINE, PAIN MANAGEMENT, Musculoskeletal disorders

## Abstract

**Introduction:**

An estimated 10% of women experience severe, chronic pelvic girdle pain post partum. This has significant physical, psychological and socioeconomic consequences. Typically, such pain is recalcitrant to conservative management; hence the need to identify effective management strategies. Customised Dynamic Elastomeric Fabric Orthoses may be an option to address this gap; designed to improve pain by providing support while optimising movement and function. Currently, no studies have evaluated the clinical and cost-effectiveness, or acceptability of these customised orthoses in postpartum women.

**Methods and analysis:**

EMaPP is a pragmatic, multicentre randomised controlled feasibility trial with an embedded qualitative study and economic evaluation. Sixty participants with pregnancy-related severe pelvic girdle pain >3 months post partum will be recruited. Participants will be randomly allocated in a 1:1 ratio (stratified by centre and presence/absence of lumbo-pelvic pain pre pregnancy) to receive either standard care (standardised information and exercise) or intervention (orthosis plus standard care). All participants will be asked to complete a battery of self-report questionnaires (including pain, function, health-related quality of life and health and social care resource use), via a web-based application at baseline, 12 weeks and 24 weeks. Pain levels and medication usage will be reported fortnightly. Feasibility and acceptability of the trial procedures will be determined in terms of recruitment and retention rates, data completion rates and intervention adherence. Five clinicians and 10 participants will be interviewed to explore their experiences of the trial procedures and receiving the intervention.

**Ethics and dissemination:**

This study was approved by: National Research Ethics Scheme (NRES Committee Health and Care Research Wales Research Ethics Committee (21/WM/0155) and University of Plymouth Faculty of Health Research Ethics and Integrity Committee (ref:2966). Results will be made available to participants, the funders, staff, general public and other researchers through a range of mechanisms.

**Trial status:**

Currently recruiting.

**Trial registration number:**

ISRCTN67232113.

**Strengths and limitations of this study**

- EMaPP is the first multicentre randomised controlled feasibility trial assessing the feasibility and acceptability trial procedures, comparing the delivery of a novel pelvic orthosis plus standard care with standard care alone in women experiencing severe pelvic girdle pain post partum.
- All trial procedures and outcome measures are undertaken virtually, examining the acceptability of this approach.
- EMaPP data collection has accounted for the worst and average pain with the Numerical Rating of Pain Scale (in full), collected fortnightly, capturing the variability and the cyclical nature of pain experienced by participants.
- The trial includes an embedded qualitative element investigating acceptability of the intervention and trial procedures, with purposive sampling to capture a diversity of views from the participants and clinicians.
- A limitation is that adherence to exercise is not systematically captured.

## Introduction

An estimated 70% of women experience pelvic girdle pain (PGP) during pregnancy,^1^ with approximately 10% of these experiencing this for longer than 3 months post partum. For some this pain is severe and long lasting, with reports of pain for more than a decade post partum.^2–4^ This has significant physical, psychological and socioeconomic consequences. Often everyday activities are affected such as moving in bed, walking, driving, breast feeding (due to discomfort and the effect of analgesia in breast milk), continence (due to associated pelvic floor dysfunction) and safely caring for the child and siblings, especially as the child becomes more mobile.^5^ The emotional impacts of ‘living with enduring pain’, include anger and frustration and feelings of ‘being a burden’,^6^ with related abuse of analgesics highlighted.^7 8^ Work absenteeism contributes significantly to the economic consequences.

Despite such wide-ranging impacts, symptoms can be overlooked or dismissed by health professionals believing the pain will naturally resolve.^2 9^ However, severe long-lasting pain is often recalcitrant to usual management,^10 11^ and can ultimately require invasive and expensive fluoroscopy guided injections and surgical procedures (ablation and fusion). There is an urgent need for effective strategies to address this personal and societal burden. In particular, guidance is needed for the extended postpartum phase since all current guidance relates to pain during pregnancy and up to 8 weeks post partum.^12^ In light of this, National Health Service (NHS) England has committed to improving access to postnatal physiotherapy.^13^

Pelvic orthoses are one option to manage this condition having support from European guidelines,^14^ and the Royal College of Obstetricians and Gynaecologists.^15^ These are externally worn devices designed to increase pelvic joint stability, musculoskeletal alignment and sensory input to optimise muscle control, pain and function.^16^ There is currently a wide range of ‘off the shelf’ pelvic orthoses available, however they have been shown to be of mixed benefit and often ineffective when pain is severe.^17^ Furthermore, women report wear-time issues due to discomfort, lack of ease of use, reduced aesthetics and impact on movement.^8^ Sufficient wear time is however crucial for orthotics as benefits gained are dependent on this.

A novel customised Dynamic Elastomeric Fabric Orthosis (DEFO) for people with PGP has been designed, (DM Orthotics’, https://www.dmorthotics.com) to address these issues. This pelvic orthosis differs markedly in the design, material and compression grades to ‘off the shelf’ pelvic orthoses. Their use has been previously assessed; during pregnancy, through a randomised controlled trial (RCT), finding that they significantly improved pain over and above the traditional rigid belt;^18^ and in a replicated case series of group of eight women experiencing postpartum PGP, identifying an improvement in pain, function and quality of life.^19^ Both studies indicated the pelvic DEFOs were acceptable to wear with regard to wear time, comfort and aesthetics. These studies together indicate a potentially effective new treatment option to managing this difficult to treat condition in the postpartum period. Further evidence is required to establish the clinical and cost-effectiveness of this novel intervention in women experiencing persistent severe PGP following pregnancy. Before considering a definitive trial, the feasibility of running such a trial needs to be tested.

### Aims and objectives

The primary aim is to assess the feasibility and acceptability of the trial procedures comparing the delivery of a novel pelvic orthosis plus standard care with standard care alone in women experiencing severe, chronic PGP post partum.

The trial objectives are to gain operational experience and gather high-quality data to inform the conduct and design of an anticipated definitive RCT and cost-effectiveness analysis, including the most appropriate primary and secondary outcome measures, the most effective recruitment methods and to inform the sample size calculation of the future trial.

## Methods

### Trial design

**Figure 1:**
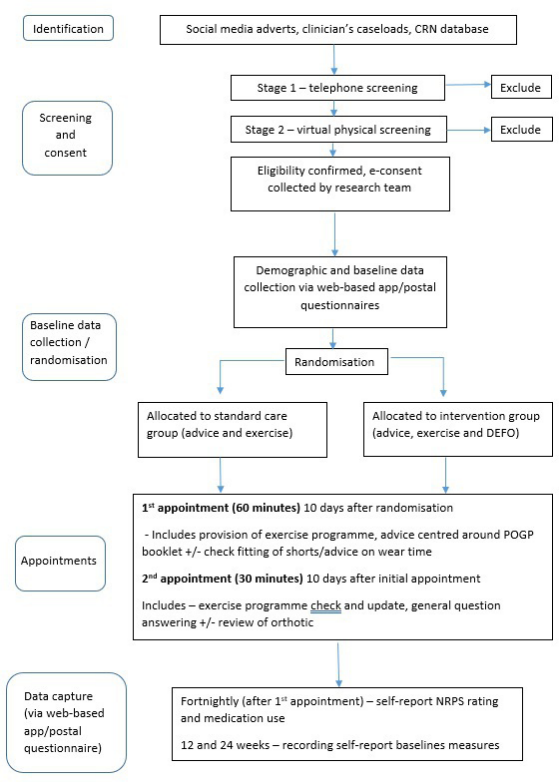
Trial flow chart. CRN, Clinical Research Network; DEFO, Dynamic Elastomeric Fabric Orthosis; NRPS, Numerical Rating of Pain Scale; POGP, Pelvic, Obstetric & Gynaecological Physiotherapy.

The EMaPP trial is an assessor blinded, pragmatic, randomised controlled feasibility trial comparing standard care (standardised advice and exercises) to standard care in addition to a customised pelvic orthosis, with an embedded qualitative study. A Consolidated Standards of Reporting Trials (CONSORT) study flowchart outlines the participant pathway through the trial (figure 1). The study started in March 2021 and ends in June 2023.

### Study settings

The three study sites are based in three geographical regions of England: Devon, West Yorkshire and Buckinghamshire. The interventions are delivered in primary or secondary care subject to local practices.

The trial undertakes a remote, distance-based approach to its delivery at all stages.

- Telephone and virtual screening.
- Interventions delivered via videoconferencing by NHS physiotherapy services using locally approved software.

To prevent digital exclusion, face-to-face intervention will be delivered at a local healthcare establishment when required.

### Eligibility criteria

The study population will comprise women with postpartum PGP, based on the eligibility criteria outlined in table 1.

**Table 1:** EMaPP study inclusion and exclusion criteria

Inclusion criteria	Exclusion criteria
Aged ≥18 years.	Known allergy to Lycra.
Able and willing to provide informed consent.	Currently pregnant.
Self-report persistent PGP (for a minimum of 3 months post partum).	Currently wearing a catheter.
Self-reported severe PGP (causing walking or stair climbing to be bothersome).	Self-reported history of pathologies causative of lumbo-pelvic pain (eg, infection, trauma, cancer).
Diagnosis of PGP in line with European guidelines; defined as pain between the posterior iliac crest and inferior gluteal fold, particularly in the sacroiliac joint vicinity, that may radiate to the posterior thigh and occur in conjunction with or separately in the symphysis pubis,^14^ captured using the pain referral map.	Participating in concurrent interventional research which may overburden the patient or confound data collection.
Scoring positively with at least one anterior PGP test and two tests for posterior PGP or two positive tests for posterior PGP^39^ (figure 3).	Participants unable to understand verbal and/or written English adequate to complete the trial procedures/self-report questionnaires. Assistance from a friend/family member for translation purposes is acceptable.
The PGP must have started or been aggravated during pregnancy, as determined by self-report.	

PGP, pelvic girdle pain.

**Figure 2:**
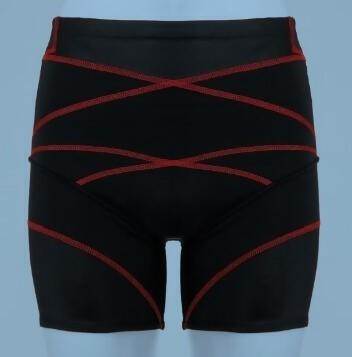
Customised pelvic support shorts (Dynamic Elastomeric Fabric Orthosis).

### Participant recruitment, identification and screening

A multifaceted recruitment approach will be undertaken as follows:

Nationally, social media will be used to promote the trial via a various sites including: Mumsnet, Facebook and Twitter pages of the pelvic, obstetric and gynaecological physiotherapy special interest group, National Maternity Voices, National Childbirth Trusts and the dedicated study website (https://www.plymouth.ac.uk/research/emapp-trial).

Locally, recruitment will occur via: the Clinical Research Networks who will undertake selected General Practitioner (GP) database searches; caseloads of physiotherapists working within musculoskeletal, women’s health services of the recruiting sites; posters in relevant outpatient clinics/ waiting areas of participating sites; local patient and public involvement and engagement (PPIE) groups and hospital social media accounts.

The trial will use a dedicated email address, included on all promotional material along with a QR code, to direct interested parties to further information on the trial website. The trial website contains a downloadable participant information sheet (PIS) and reply slip.

All interested participants, and those on databases or caseloads who are deemed potentially eligible, will be sent a trial information pack containing: PIS; details of the ethically approved study sites; and a reply slip to indicate their interest and confirm they feel they are eligible.

### Eligibility screening process

Eligibility screening will occur via a two-stage process. On receipt of the completed reply form a telephone screen (stage 1) will be undertaken using a pre-formatted screening checklist based on the eligibility criteria. If deemed eligible, a video conference physical screening assessment will be arranged (stage 2). A screening pack will be sent out ahead of this physical screening appointment containing: the screening battery tests (schema of physical tests and instructions), Beighton self-report score, trial consent forms, orthotic measuring guide and tape measure (to capture measurements for production of the customised orthosis, should the participant be allocated to the intervention group) and digital callipers.

The physical screening requires the presence of a partner/trusted other to assist with the physical assessment and orthosis measurements, under the guidance of the remote researcher. All screen failures will be recorded.

### Randomisation

Once eligibility is confirmed, the consent process completed, and screening data entered into the trial database, participants will be sent a text/email, generated by the Peninsula Clinical Trials Unit (PenCTU), requesting them to complete the baseline questionnaires. Once complete, the participant will be randomly allocated on a 1:1 ratio, using random permuted blocks, stratified by centre and presence/absence of lumbo-pelvic pain pre-pregnancy, to either the standard care or intervention group (figure 1). Screening data capture and randomisation is achieved via a web-based system created by the PenCTU.

After randomisation, a series of emails will be automatically generated. A blinded email will be sent to the research team members involved in eligibility screening. An unblinded email will be sent to the chief investigator (CI), study administrator and site principal investigator (PI). An email/text will be sent to the participant to inform them of their group allocation.

### Blinding

The trial participants and NHS treating physiotherapists are unable to be blinded due to the nature of the intervention. Every effort will be made to ensure the research team is blinded to group allocation throughout the trial. The statisticians will remain blind to group allocation when conducting the primary analyses until analyses that necessitate unblinding need to be performed, such as wear-time analysis. The research team will keep a detailed log of any inadvertent unblinding events. Final unblinding of the research team will occur after database lock and blinded analysis has been undertaken.

### Interventions

NHS physiotherapists will provide all interventions at the two protocolised video-based sessions. At the participant’s preference, face-to-face sessions can be undertaken in place of these remotely delivered sessions. The initial session will last a maximum of 60 min and the follow-up session (a maximum of 30 min) will be scheduled approximately 10 days later. Participants are not prevented from receiving other care. This will be captured in the health resource use questionnaire.

#### Standard care group (standardised information and exercise)

The standard care regime was designed in collaboration with women’s health physiotherapists experienced in the treatment of persistent PGP. This was considered by them to be reflective of usual clinical practice. It comprises two components:

##### Standardised information

This will be provided through a discussion centred on the publicly available booklet produced by the pelvic, obstetric and gynaecological physiotherapy special interest group ‘Guidance for mothers-to-be and New Mothers: Pregnancy-related Pelvic Girdle pain’ (https://pogp.csp.org.uk/system/files/pogp-pgppat_3.pdf).

##### Standardised exercise

The physiotherapist will teach participants a programme of up to four lumbo pelvic/hip exercises, typical of those provided within usual physiotherapy practice. These will be selected from a protocolised set (online supplemental file 1), on the basis of their initial assessment. Participants will be advised to complete the exercises on 3 days of the week, with each session lasting approximately 20–30 min. All participants will receive an exercise leaflet describing the exercises, with accompanying images. This will be posted or emailed dependent on participant preference.

10.1136/bmjopen-2022-063767.supp1 Supplementary data

#### Intervention group (standardised information and exercise plus DEFO)

In addition to standard care, participants allocated to the intervention group will be provided with two pairs of customised DEFOs (figure 2, DM Orthotics, https://www.dmorthotics.com). The orthosis will be worn for the first time at the initial intervention session, enabling the physiotherapist to check the fit; this will also be reviewed at the follow-up session. Participants will be advised to wear the DEFO for a maximum of 12 hours per day, with wear time graduated over the course of the first 5 days in line with manufacturer guidance. The orthoses are delivered with an advice booklet detailing donning and doffing, wear time and care instructions, with a supplementary video hosted on the trial web page. Where alterations are required because of poor fit, in line with current practice the NHS physiotherapists will follow a standard operating procedure, making contact with the orthotic company to arrange an adjustment if required.

**Figure 3:**
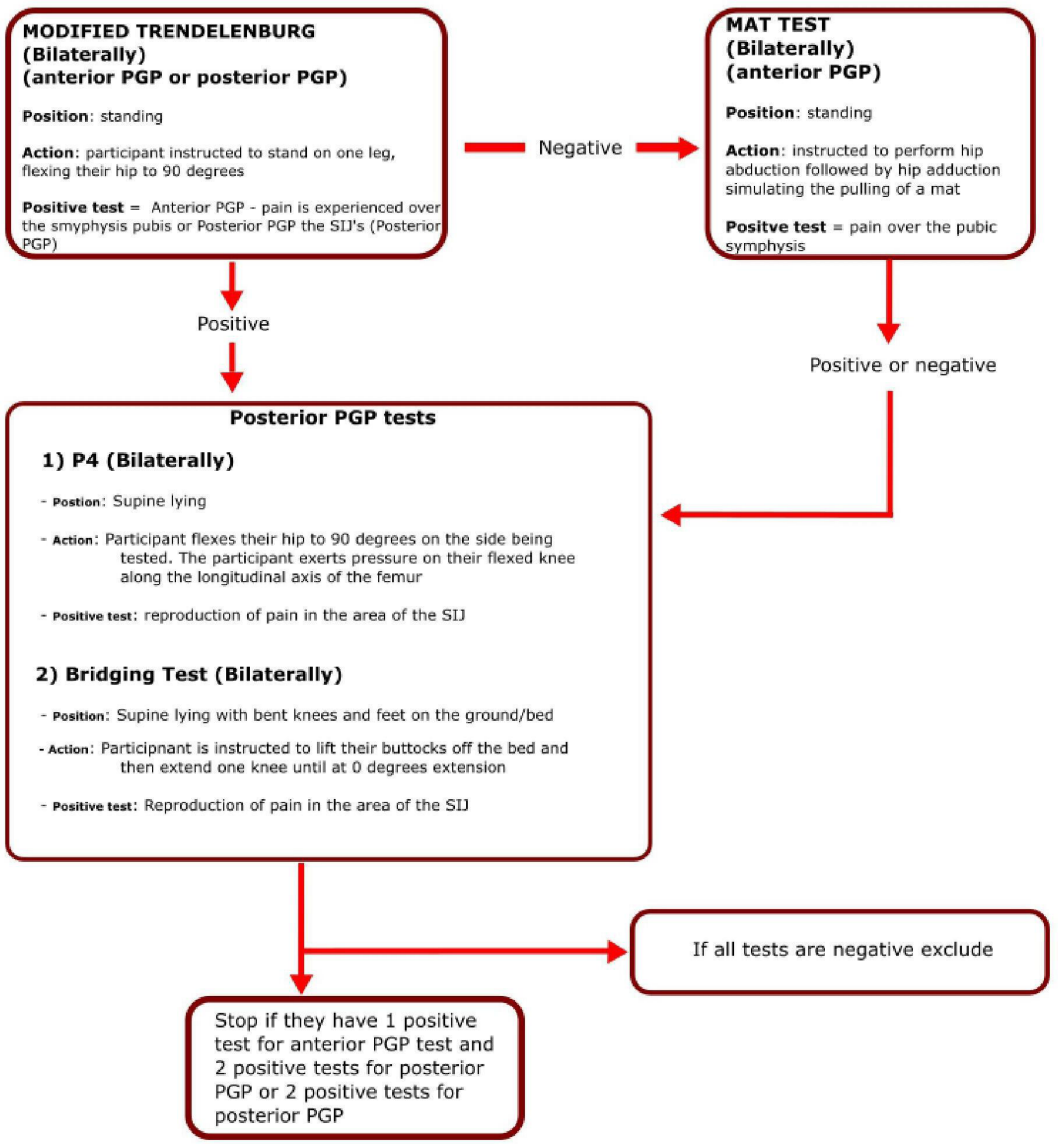
Pelvic girdle pain screening battery. PGP, pelvic girdle pain; SIJ, Sacro Iliac Joint.

### Data collection

#### Feasibility outcomes

The trial outcomes include feasibility and acceptability of the EMaPP trial. Quantitative and qualitative feedback will be obtained to identify the main determinants of experience and acceptance of the trial in line with the trial objectives. In particular, the following measures and operational criteria will be determined:

- Number and proportion of eligible participants from the target population.
- Willingness of participants to be randomised.
- Recruitment and retention rates of eligible participants through the trial.
- Completeness of data sets/outcome measures.
- Incidence of face-to-face sessions.

#### Clinical outcome measures

A battery of standardised, patient reported outcome measures, together with demographic and diagnostic characteristics (birth details, pain history, Beighton Score^20 21^ and two point estimation (2PE) Task,^22 23^ will be collected at baseline. A selection of outcome measures will be repeated fortnightly and at 12 and 24 weeks after the first-intervention session to map in more detail the trajectory of pain over the trial timeline (table 2). All patient reported outcome measures will be reported directly by the participants via the web-based application. At each time point, participants will automatically receive five text/email reminders (one per day) if the data entry has not started. All patient reported outcome measures included within this study align with the PGP core outcome set for evaluating the effectiveness of interventions in postpartum PGP.^24^

**Table 2:** Assessment schedule

Assessments	Baseline	Post-initial appointment		
		Fortnightly	12 weeks	24 weeks
Demographics, birth details, pain history	x			
Beighton score^20 21^ to determine joint hypermobility	x			
Two-point estimation task^22 23^ to determine implicit somatoperception	x			x
Pain medication use	x	x	x	x
Numerical Pain Rating Scale^25 26^	x	x	x	x
Health and Social Care Resource use Questionnaire to collect data on health, social and wider care resource use^18^	x		x	x
Edinburgh Post-natal Depression Scale^33^ to assess post-natal depression (cut-off **≥**12 points)	x		x	x
Freemantle Back Awareness Questionnaire to assess explicit body perception of the lumbo-pelvic region^36 37^	x		x	x
Pelvic Girdle Questionnaire^40^ to assess function and symptoms	x		x	x
Short-form 36^41^ to assess health-related quality of life and calculate quality adjusted life-years (QALYS)	x		x	x
EQ-5D-5L to assess health-related quality of life and calculate QALYS^42^	x		x	x
International Consultation on Incontinence Questionnaire, Urinary Incontinence—Short Form to assess level and impact of symptoms of incontinence^43^	x		x	x
Tampa Scale Kinesiophobia to assess fear of movement/kinesiophobia^44^	x		x	x
Wear-time adherence (Orthotimer)				x
Qualitative interviews				x

#### Proposed clinical outcome measures

Numerical Rating of Pain Scale (NRPS) is the proposed primary outcome measure for the definitive RCT. This 0–10 point scale is widely used,^25^ quick to complete and with a format suitable for completion through a mobile/application. There is evidence in chronic pain patients to suggest a one-point change is clinically significant,^26^ and hence a difference between groups of >1.0 at 24-week follow-up will indicate a signal of efficacy. Participants will rate their pain experience over the past fortnight using the NPRS within four categories: (i) worst level of day-time pelvic pain, (ii) average level of day-time pelvic pain, (iii) worst level of night-time pelvic pain, (iv) average level of night-time pelvic pain.

The proposed secondary outcome measures are detailed in table 2.

Resource use and costs associated with delivery of both intervention arms will be estimated. Resource use associated with the measurement, ordering, delivery and production of the orthosis and the physiotherapy interventions, will be collected via within trial reporting, including participant level contact and non-contact time for NHS staffing input.

### Intervention fidelity

NHS clinicians will complete a pre-formatted intervention fidelity checklist for each of the two physiotherapy sessions. Adherence to wearing the orthosis will be assessed via a temperature sensor integrated into the seams of the orthosis. The Orthotimer sensor (Rollerwerk Medical Engineering, Balingen, Germany) is embedded in a small (13 mm×9 mm×5 mm), dust and watertight unit. It will record time, date and temperature every 15 min over the 24-week period with temperature precision of ±0.1°C. DM Orthotics will activate and sew the sensor into each orthosis before dispatching to participants. Adherence to the home exercise programme is not assessed during the trial, however it is explored via the qualitative interviews.

#### Adverse events

Throughout the trial, all possible precautions will be taken to ensure participant safety and well-being. Participants will be monitored for adverse events throughout the 24 weeks. The likelihood of participants being harmed by either wearing the orthosis or undertaking the exercise programme (which reflects usual clinical practice) or any of the trial procedures is considered very low. Collection and reporting of adverse events will be captured via the web-based app and relate to those classified as an adverse reaction (including adverse device effects) or those which are serious. These will be collected at fortnightly intervals. On receipt of a reported reaction or serious adverse event, the CI will contact the local PI to discuss causality. All adverse events will be reported at the Trial Steering Committee (TSC) and Trial Management Group (TMG) meetings.

#### Qualitative substudy

The aim is to explore the experiences of wearing (participant) and providing (clinicians) the pelvic orthosis and engaging in the trial procedures.

The specific objectives are to investigate acceptability of the trial methods across both arms, acceptability (comfort, wear time) of the orthosis, impact the intervention may/may not have on the participant’s life and adherence to the exercise regime.

Semi-structured telephone interviews will be undertaken, at the end of the trial, with 10 purposively sampled participants (n=5 intervention group, n=5 standard care group) and five NHS physiotherapists involved in delivering the interventions. A topic guide will be used (online supplemental file 2). Interviews will be digitally recorded and transcribed verbatim.

10.1136/bmjopen-2022-063767.supp2 Supplementary data

Informal telephone exit interviews will be undertaken with participants that withdraw from the trial to share their reason for withdrawing. The interviews will not be recorded or transcribed. Field notes will be taken.

#### End of trial definition

There are no formal stopping criteria. It will be prematurely stopped on safety grounds through a decision by the TMG and TSC.

#### Sample size

As this is a feasibility trial, a formal sample size calculation, based on power for detecting a clinically meaningful between-group difference in a primary clinical outcome, will not be undertaken. The trial aims to recruit 60 participants over a 7-month period. The sample size is pragmatic and determined to be large enough to provide robust estimates on the likely recruitment rates and follow-up and estimates of the variability on the proposed primary outcome to inform a future sample size calculation. The target is set to enable determination of whether it would be practicable to recruit adequate numbers in a manner conducive to implementing a reasonably costed definitive trial. A sample size of 60 will allow overall retention rate at 6-month follow-up to be estimated to within a 95% CI of approximately ±13% (±10% if retention rate is 80%). Assuming a non-differential retention rate of 80%, this should provide follow-up outcome data on a minimum of 24 participants per group.

### PPIE

Women with experience of severe chronic PGP provided input into the study design. Priority of the research question, choice of outcome measures, recruitment methods and mode of intervention delivery were informed by discussions with women through a focus group session and ongoing discussions with a dedicated lay member of the project team. Lay input continues through membership on the TMG, and an additional independent lay member on the TSC. PPIE will play a key role in the dissemination of trial findings, contributing to and reviewing material.

### Determining progression to the definitive trial

Progression to a full trial application will occur if minimum success criteria are achieved in key feasibility aims and objectives, and/or if solutions can be identified to overcome any issues. These criteria will be finalised in discussion with the TSC. A red, amber, green system will assist the decision-making progress, with red indicating ‘Stop’, Amber indicating ‘Discuss and Modify’ and green indicating ‘Go’. The anticipated progression criteria are detailed in table 3.

**Table 3:** Progression criteria

Criteria	RAG rating
60 participants recruited within a 7-month recruitment window.	Red: <60% Amber: 60%–80% Green: >80%
Percentage of participants randomised to intervention group non-compliant in wearing the shorts (non-compliance—wearing the shorts for less than 6 hours/day or total of 42 hours/week).	Red: >70% Amber: 50%–70% Green: <50%
Percentage of participants completing primary outcome measure (NRPS) at 24-week follow-up.	Red: <60% Amber: 60%–80% Green: >80%
*Percentage of participants completing secondary outcome measures at 24-week follow-up in (in the following order of priority (EQ-5D-5L, SF-36, PGQ, ICIQ).	Red: <60%, Amber: 60%–80%, Green: >80%
Evidence to suggest efficacy, that is, that the support shorts hold promise as an effective intervention, demonstrated by an 80% CI that indicates plausibility of the between group difference in the primary outcome measure being ≥1 point, on the NRPS.	N/A
Total resource estimated to conduct the definitive trial within levels likely to attract funding.	N/A

*This progression criteria relates to the selection of outcome measures to be used in a definitive trial. It does not influence the decision as to whether or not there should be progression to a definitive trial.

CI, confidence interval; ICIQ, International Consultation on Incontinence Questionnaire; NRPS, Numerical Rating of Pain Scale; PGQ, Pelvic Girdle Questionnaire; SF-36, Short Form-36.

## Ethics and dissemination

The EMaPP trial has received approval from the: Research Ethics Committee (REC) (REC reference number: 21/WM/0155); Health Research Authority (HRA); and confirmation of capacity and capability from the Research and Development departments of the participating centres. Any amendments to the protocol will be discussed with the sponsor and submitted to HRA/REC for approval.

Dissemination will target users, clinicians and researchers. The results will inform the design of the anticipated definitive trial. It will not inform clinical decision-making since clinical and cost-effectiveness cannot be determined at this level. Dissemination, regardless of outcome will be via: publication of results in a peer-reviewed journal, a funding proposal for a full scale effectiveness trial (if progression criteria are met), lay orientated research feedback events and a lay summary for trial participants, staff and the general public and via conference presentations at grass roots physiotherapy events and research conferences. The study report will be accessible on the study web page (https://www.plymouth.ac.uk/research/emapp-trial), as will the trial protocol and statistical analysis plan. The protocol has been written and published in line with Standard Protocol Items: Recommendations for Interventional Trials guidelines.^27^ Publications will follow CONSORT guidance for feasibility trials,^28^ and the template for intervention description and replication) guidelines.^29^ Authorship of the intended articles will be the study team; professional writers will not be used.

### Changes post-trial registration

Initial inclusion criteria included a ceiling to pain chronicity of 2 years. This was amended to include women with pain persistence of >2 years, following discussion with the trial steering committee, to optimise recruitment. In addition, an ethical amendment was approved for the clinical care team to contact women who had been sent a PIS but had not completed the attached reply slip; to understand the reasons behind this.

### Economic evaluation

The resources required to provide the intervention will be assessed and a framework established for a future cost-effectiveness analysis alongside a definitive RCT. Data on intervention resources will be collected via within-trial reporting, including participant-level contact and non-contact time and training for delivery staff. Participants will self-report health, social and wider care resource use, using the Resource Use Questionnaire. Participants will complete the EQ-5D-5L (the anticipated primary economic outcome measure in a full trial), and quality adjusted life-years (QALYs) will be estimated over the follow-up period. The appropriateness of the Short Form-6 Dimension (SF-6D), based on the Short Form-36 (SF-36) for estimating QALYs will be explored. The economic evaluation methods will be developed to provide a future policy-relevant cost-effectiveness analysis of the intervention in the context of the UK NHS/Personal Social Services.

### Data management and analysis

Detailed data management activities are described in a separate data management plan. PenCTU staff will monitor completeness and quality of data throughout the trial. No imputation of missing data will be undertaken.

#### Quantitative

A detailed statistical analysis plan will be finalised ahead of trial database locking. A CONSORT diagram will display data from screening, recruitment, consent and follow-up logs and be used to generate estimates of eligibility, recruitment, consent and follow-up rates. Completion rates will be estimated for outcome measures at each time point, including the health, social and wider care resource-use data. Recruitment and retention rates will be accompanied by 95% CIs, to inform assumptions for planning the definitive trial. Adherence data (wear time) will contribute to evaluation of intervention acceptability/feasibility and evaluated as total hours and as a percentage of total possible daytime wearing time (12 hours/day). The percentage of week’s participants wore the shorts for a minimum of 42 hours a week, and the percentage of days participants wore the shorts for a minimum of 6 hours a day, will be summarised.

All outcomes will be summarised by allocated group at each follow-up with appropriate descriptive statistics. Between-group differences will be reported with 80% CIs in addition to 95% CIs, with particular focus on change in pelvic girdle day-time pain at the primary end point of 24 weeks. According to the 2008 Initiative on Methods, Measurement, and Pain Assessment in Clinical Trials (IMMPACT) consensus statement,^26^ changes of one point represent minimally important decreases in pain using the NRPS of pain intensity. We will inspect the CI of the between group difference to see if there is evidence of a plausible signal of efficacy. Estimates of the variability of outcome measures and the correlation between baseline and follow-up outcome measures will inform the sample size calculation for a future definitive RCT.

#### Qualitative

Thematic analysis will be used for the telephone interview data using NVivo V.12 software for organisation and analysis (QSR International, Southport, UK, July 2021). Braun and Clarke’s six-phase process will be used: (i) data familiarisation; (ii) coding; (iii) generating initial themes; (iv) reviewing themes; (v) defining and naming themes and (vi) writing up to identify patterns of meaning within the data sources. Initial themes will be refined by two researchers to maximise credibility and dependability.

Interviewees will be invited to review a draft of the analysis to ensure accurate representation of their views/experiences.

## Discussion

This is the first RCT to explore the feasibility and acceptability of a novel customised pelvic orthosis in the management of chronic PGP in the postpartum period. There is a lack of evidence to support the management of this condition beyond the first 8 weeks post partum. This orthosis has already shown promise as an intervention during pregnancy,^18^ and in a replicated case series study of eight women post partum.^19^ This trial aims to serve as a starting point to investigate the effectiveness of this orthosis in the management severe chronic PGP post partum.

Some aspects of the study design are noteworthy. All trial procedures and intervention delivery are undertaken remotely; described as a virtual trial.^30 31^ This replicates current practice, mitigates the risk associated with face-to-face appointments during the COVID-19 pandemic and ensures trial delivery in the event of a lockdown. It is also a potentially more efficient approach.^31^ Because some participants may not be able to participate in a fully virtual trial, to optimise generalisability provisions have been made to provide a non-virtual format when this is preferred by the participant. Monitoring of the proportion of women who choose a non-virtual approach will further inform the future trial design.

All outcome measures collected at follow-up time points (apart from adherence to wear time in the intervention group) are patient reported outcome measures (PROMs), and via a web-based application. This approach was chosen on the basis of PPIE input, the rationale being to minimise participant burden. The collection of PROMs minimises the influence of the research team on data collection. Recommendations from Mercieca-Bebber,^32^ have been used to improve PROMS data collection during this trial, such as reducing free-text options in questionnaires, flexible data entry and text/email reminders to enhance data completeness. With regard to wear-time adherence data, this too is collected remotely using the Orthotimer temperature sensor; with the added advantage of providing detailed and objective data which is otherwise challenging to reliably collect via methods such as patient diaries.

The qualitative interviews are also undertaken via remote means. This decision was informed by PPIE discussions, aiming to reduce participant burden in terms of travel time and inconvenience; particularly important considerations for people in pain and looking after young children.

The self-report depression questionnaire, Edinburgh Postnatal Depression Scale (EPDS)^33^ is used to assess post-natal depression. If a score of >12 points (indicative of depression) is recorded, an email to the research team is triggered to contact the participant and their GP, thereby to ensuring duty of care is upheld. The data collected will have the added advantage of increasing understanding of the prevalence of post-natal depression in this population; currently an under-researched area.

Persistent PGP is difficult to manage with the aetiology of the condition,^34 35^ and the mechanism of action of pelvic orthotics under debate. One factor that has recently been under the spotlight within this group and may represent a possible mechanism of action for an orthotic, relates to changes in somatoperception (the perception of size and shape), with evidence pointing to an altered explicit somatoperception in both the peri-natal^36^ and post-natal period.^37^ This study has included explicit (via the Freemantle Back Awareness Questionnaire (FreBAQ)) and implicit (measure of 2PE) measures of somatoperception, as recommended by Viceconti *et al*.^38^ It is the first to study both types of somatoperception in parallel during an interventional trial.

## Supplementary Material

Reviewer comments

Author's manuscript
