# Supplementary material for: Evaluating the Management of chronic Pelvic girdle Pain following pregnancy (EMaPP): study protocol for a randomised controlled feasibility trial to compare a customised pelvic orthosis with standard care
Source: BMJ Open. 2022 Aug 4;12(8):e063767. doi: 10.1136/bmjopen-2022-063767 (PMC9361753; doi:10.1136/bmjopen-2022-063767)
Supplement: Supplementary data [file bmjopen-2022-063767supp001.pdf]

Supplementary file 1: Standardised Exercise regime

Reference: Exercises adapted from "Exercise and Advice after pregnancy" PGOP booklet. https://pogp.csp.org.uk/system/files/publication_files/POGP-E%26AA5%28UL%29.pdf

Location: completed at home independently.

Frequency: Aim x 3 per week

Dosage: Aim for up 20 - 30 min's per session. Participants may need to break this into 2 parts.

Repetitions: Exercises described have suggested sets and repetitions. However, this should be guided by treating clinician and the women's capacity to exercise.

Quantity: Choose a maximum of 4 exercises

Advice to be provided to all women: If any of the exercises cause pain then reduce the amount of repetitions that you are performing. If this continues on more than two occasions, then cease the exercise and discuss with your clinician at your follow up appointment.

Permitted exercises are described below:

1) Pelvic floor exercises
   - *Exercise description*: Imagine that you are trying to stop yourself passing urine or wind: 'squeeze and lift' your pelvic floor muscles, closing and drawing up the 3 passages. You may not feel that much is happening at first but keep trying. Hold the squeeze for a few seconds, and then relax for a few seconds. Do not hold your breath. You may find that abdominal hollowing takes place at the same time.
   - *Positions* – consider the use of different position to exercise in (e.g. standing, sitting and lying),
   - *Speed of contraction/Dosage*:
     - *For endurance:* Start gently and rhythmically. Gradually increase the hold time and the number that you do until you can hold the squeeze for up to 10 seconds and repeat up to 15 times in a set (up to 3 sets)
     - *For speed*: It is also important that the pelvic floor muscles are able to react quickly to stop you leaking when you cough, sneeze, laugh or shout. Tighten them as quickly and strongly as you can, and then relax. Do this between 8-15 times a set with up to 3 sets.
   - *Advice*: Always tighten your pelvic floor muscles before and during any activity requiring physical effort; for example, when lifting, coughing or sneezing

2) Abdominal hollowing exercise:
   - *Exercise description*: Place one or both hands on your abdomen, below the level of your umbilicus ('tummy button'). Breathe in through your nose, and as you breathe out, draw in your lower abdomen, pulling it gently away from your hands towards your back and then relax. You should be able to breathe and talk while you are doing this exercise and your lower back should stay still. You may feel the muscles working under your hand.
   - *Positions*: Start doing this exercise in the most comfortable position for you; for example, lying on your back or side with your knees bent, or sitting with your back well supported. Once you have learnt this exercise, you should be able to practise it in standing.

- Suggested Dosage: Start at a level that your patient can tolerate. Aiming to reach a hold 10 seconds and can repeat for a maximum of 10 times up to 3 sets.
- *Advice*: You may also feel these muscles working as you do the pelvic floor muscle exercises. Use these deep muscles by doing the hollowing throughout the day - before and during any physical activity requiring effort; for example, when lifting. If you notice any bulging of your abdominal muscles while doing any of the following 4 exercises, stop the exercise, return to abdominal hollowing and seek advice from your physiotherapist.

3) Pelvic tilt**:**
   - *Exercise description*: Hollow your abdomen as described above, tighten your pelvic floor muscles, and flatten your lower back into the bed as your pelvis tilts. Breathe normally. Hold the position for 3 seconds and release gently. Repeat this up to 8-12 reps for 3 sets.
   - *Position*: Progress by doing this exercise when in sitting, standing, crook lying, kneeling or lying on your side.
   - Suggested Dosage: Aim for between 8-15 reps for up to 3 sets.

4) Bridge:
   - *Exercise description*: Place both feet flat on the bed/floor. Hollow your abdomen as described above, tighten your pelvic floor muscles. Slowly lift your bottom off the bed to a height you are comfortable with.
   - *Position*: Crook lying
   - Suggested Dosage: Hold for up to 3 seconds before slowly lowering. Aim to complete 8-15 for up to 3 sets.

5) Knee bends:
   - *Exercise description*: Hollow your abdomen, keep your back flat on the bed, and bend one hip and knee up as far as is comfortable. Hold up to the count of 10, and then bring the leg down so that the foot is back on the bed. Repeat with the other side.
   - *Position*: crook lying
   - Suggested Dosage: Hold for 10 seconds. Aim for 8-15 reps for up to 3 sets.

6) Knee rolls:
   - *Exercise description*: Hollow your abdomen, and gently lower both knees to the right as far as is comfortable. Bring them back to the middle and rest for a few seconds. Hollow your abdomen again and repeat to the left.
   - *Position*: crook lying
   - Suggested Dosage: Aim for between 8-15 reps for up to 3 sets.

7) Head lift:
   - *Exercise description*: Lie on your back with your head resting on two pillows. Hollow your abdomen and tighten the pelvic floor muscles as you gently lift your head and shoulders a little way off the pillows.
   - Position: crook lying

- Advice: If you have neck pain, DO NOT do this exercise.
- Suggested Dosage: Hold for 3 seconds, lower and relax. Repeat between 8-15 reps for up to 3 sets.
- Progression advice: Progress by removing one pillow and ensure that you do not strain your neck muscles as you do this exercise.

8) Hip extension:
   - *Exercise description*: Participant lies prone on a flat surface with both hips in neutral and knees in full extension. Action: Hip extension against gravity
   - *Positions*: in standing with a flexed knee or in prone lying
   - *Suggested Dosage*: Aim for between 8-15 reps for a maximum of 3 sets with guidance on adding a weight or theraband as able.

9) Hip Abduction:
   - *Exercise description*: Side lying hip abduction: In a side lying position the participant abducts superficial limb to their maximum height, slowly lowers the limb back to its resting position.
   - Positions: Side lying
   - *Suggested Dosage*: Aim up to 3 sets of 8-15 reps. Position of knee bend is at therapist discretion. Perform either at 90^0 knee flexion or 0^0 extension

10) Knee Drop outs:
   - Exercise description: Hollow your abdomen, keep your back flat on the bed and feet in contact with the bed. Allow one leg at a time to drop away from your midline down towards the surface you are lying on as far as you feel able before returning back to the starting position
   - Positions: Crook lying
   - Suggested Dosage: Aim for between 8-15 reps over 3 sets.
