# Supplementary material for: Evaluating the Management of chronic Pelvic girdle Pain following pregnancy (EMaPP): study protocol for a randomised controlled feasibility trial to compare a customised pelvic orthosis with standard care
Source: BMJ Open. 2022 Aug 4;12(8):e063767. doi: 10.1136/bmjopen-2022-063767 (PMC9361753; doi:10.1136/bmjopen-2022-063767)
Supplement: Supplementary data [file bmjopen-2022-063767supp002.pdf]

Supplementary file 2: Qualitative interview topic guide

Introduction

- Thank you for taking part in this interview (Post EMaPP trial Qualitative interview or exit interview)–
- We would like to check that you have had the opportunity to read the participant information sheet provided for this part of the study
- Check that consent form is signed
- I have some questions that we would like to ask you but feel free to ask any questions at any stage.
- Recording – This meeting will be recorded if you have consented and agreement to making notes (for informal exit interview)

Women (intervention arm):

- Please can you tell me about your experience of being involved in the trial?

- How was your experience of wearing the shorts and advice/exercise?

Women (Control arm):

- Did the treatments meet your expectations?

- How was the experience of being randomised to the control arm?

Women both arms

- How did you find the data collection methods of the trial?

  Self-report measures,

  Twice weekly pain report measures,

  Text reminders,

  Virtual nature of the trial

- How did you find the remote based appointments?

  Were there any technical difficulties?

  If so was there anything that would have helped?

- Did you feel able to adhere to the exercise prescription from the physiotherapist?
    - Would there be anything that would facilitate you adherence?
    - Were there any barriers to exercise adherence?
    - If so what were these?

- Did the treatments have any impact on you?
    - Behaviour: activity level, function, posture, sleep
    - Health: pain levels
    - Psychological: mood, energy

- Final thoughts: Do you have any final points that you would like to discuss or that you feel you didn't have the opportunity to say?

Clinicians

- How did you find the process of being involved in the trial?
- How did you find process of delivering the intervention (both pelvic support shorts & exercise/advice)?
- Did the trial interventions (advice/exercise) resemble usual clinical practice?
- Is there anything else that you feel would have improved the trial?
- Final thoughts: Do you have any final points that you would like to discuss or that you feel you didn't have the opportunity to say?

Exit interviews:

What were the reason/s why you could not continue to participate with the trial?

How did you find participating in the trial?

Was there any part of the trial that if altered or improved would have led you to remain in the trial?
