# Supplementary material for: Evaluating the Management of chronic Pelvic girdle Pain following pregnancy (EMaPP): study protocol for a randomised controlled feasibility trial to compare a customised pelvic orthosis with standard care
Source: BMJ Open. 2022 Aug 4;12(8):e063767. doi: 10.1136/bmjopen-2022-063767 (PMC9361753; doi:10.1136/bmjopen-2022-063767)
Supplement: Author's manuscript [file bmjopen-2022-063767.draft_revisions.pdf]

BMJ Open is committed to open peer review. As part of this commitment we make the peer review history of every article we publish publicly available.

When an article is published we post the peer reviewers' comments and the authors' responses online. We also post the versions of the paper that were used during peer review. These are the versions that the peer review comments apply to.

The versions of the paper that follow are the versions that were submitted during the peer review process. They are not the versions of record or the final published versions. They should not be cited or distributed as the published version of this manuscript.

BMJ Open is an open access journal and the full, final, typeset and author-corrected version of record of the manuscript is available on our site with no access controls, subscription charges or pay-per-view fees (http://bmjopen.bmj.com).

If you have any questions on BMJ Open's open peer review process please email info.bmjopen@bmj.com

BMJ Open

Evaluating the Management of chronic Pelvic girdle Pain following pregnancy (EMaPP): Study protocol for a randomised controlled feasibility trial to compare a customised pelvic orthosis with standard care.

Journal:	*BMJ Open*
Manuscript ID	bmjopen-2022-063767
Article Type:	Protocol
Date Submitted by the Author:	11-Apr-2022
Complete List of Authors:	Halliday, Bradley; University of Plymouth, Faculty of Health<br>Chatfield, Sarah; University of Plymouth, Faculty of Health<br>Cameron, Lee; Aneurin Bevan Health Board<br>Carter, Kirsty; Cornwall Partnership NHS Foundation Trust<br>Hawton, Annie; University of Exeter, Health Economics Group<br>Hayward, Christopher; University of Plymouth, Peninsula Clinical Trials Unit<br>Hosking, Joanne; University of Plymouth, Peninsula Clinical Trials Unit<br>Shawe, Jill; Royal Cornwall Hospitals NHS Trust; University of Plymouth, Faculty of Health<br>Freeman, Jennifer; University of Plymouth, Faculty of Health
Keywords:	REHABILITATION MEDICINE, PAIN MANAGEMENT, Musculoskeletal disorders < ORTHOPAEDIC & TRAUMA SURGERY

SCHOLARONE™
Manuscripts

Title: **Evaluating the Management of chronic Pelvic girdle Pain following pregnancy (EMaPP): Study protocol for a randomised controlled feasibility trial to compare a customised pelvic orthosis with standard care.**

Corresponding Author: Bradley Halliday, School of Health Professions, Faculty of Health, University of Plymouth, Derriford Road, PL6 8BH. E-mail: bradley.halliday@plymouth.ac.uk

Co-authors:

Sarah Chatfield (MSc), Faculty of Health, University of Plymouth, Plymouth, England

Lee Cameron (PhD), Aneurin Bevan University Health Board, Gwent, Wales

Kirsty Carter BA (Hons), Cornwall Partnership NHS Foundation Trust, Cornwall, England

Annie Hawton (PhD), Health Economics Group, University of Exeter Medical School, Exeter, England

Chris Hayward (BSc), Peninsula Clinical Trials Unit, Plymouth, England

Joanne Hosking (PhD), Peninsula Clinical Trials Unit, Plymouth, England

Jill Shawe (PhD), Faculty of Health, University of Plymouth, Plymouth, England & Royal Cornwall Hospital NHS trust, Cornwall, England

Jennifer Freeman (PhD), Faculty of Health, University of Plymouth, Plymouth, England

Word count: 4082

For peer review only

For peer review only - http://bmjopen.bmj.com/site/about/guidelines.xhtml

ABSTRACT

Introduction: An estimated 10% of women experience severe, chronic pelvic girdle pain post-partum. This has significant physical, psychological and socioeconomic consequences. Typically, such pain is recalcitrant to conservative management; hence the need to identify effective management strategies. Customised Dynamic Elastomeric Fabric Orthoses (DEFOs) may be an option to address this gap; designed to improve pain by providing support whilst optimising movement and function. Currently, no studies have evaluated the clinical and cost effectiveness, or acceptability of these customised orthoses in post-partum women.

Methods and analysis: EMaPP is a pragmatic, multi-centre randomised controlled feasibility trial with an embedded qualitative study and economic evaluation. Sixty participants with pregnancy related severe pelvic girdle pain > 3 months post-partum will be recruited. Participants will be randomly allocated in a 1:1 ratio (stratified by centre and presence/absence of lumbo-pelvic pain pre pregnancy) to receive either standard care (standardised information and exercise) or intervention (orthosis plus standard care). All participants will be asked to complete a battery of self-report questionnaires (including pain, function, health-related quality of life and health and social care resource use), via a web-based application at baseline, 12 weeks and 24 weeks. Pain levels and medication usage will be reported fortnightly. Feasibility and acceptability of the trial procedures will be determined in terms of recruitment and retention rates, data completion rates, and intervention adherence. Five clinicians and ten participants will be interviewed to explore their experiences of the trial procedures and receiving the intervention.

Ethics and dissemination: This study was approved by: National Research Ethics Scheme (NRES Committee Health and Care Research Wales REC (21/WM/0155) and University of Plymouth Faculty of Health Research Ethics and Integrity Committee (ref:2966). Results will be made available to participants, the funders, staff, general public and other researchers through a range of mechanisms.

Trial registration: ISRCTN67232113. Registered on 5 August 2021

Trial status: currently recruiting

Key words: Pelvic girdle pain, post-partum, Dynamic Elastomeric Fabric Orthosis, pelvic orthosis, exercise, advice, feasibility trial

For peer review only - http://bmjopen.bmj.com/site/about/guidelines.xhtml

STRENGTHS AND LIMITATIONS

- EMaPP is the first multi-centre randomised controlled feasibility trial assessing the feasibility and acceptability trial procedures, comparing the delivery of a novel pelvic orthosis plus standard care with standard care alone in women experiencing severe PGP post-partum
- All trial procedures and outcome measures are undertaken virtually, examining the acceptability of this approach.
- This study will assess the resources required and establish a framework for a future cost effectiveness analysis alongside a definitive trial.
- This feasibility study is not designed to find difference in outcomes.

For peer review only

For peer review only - http://bmjopen.bmj.com/site/about/guidelines.xhtml

INTRODUCTION

An estimated 70% of women experience pelvic girdle pain (PGP) during pregnancy,[1] with approximately 10% of these experiencing this for longer than three months post-partum. For some this pain is severe and long-lasting, with reports of pain for more than a decade post-partum.[2-4] This has significant physical, psychological and socioeconomic consequences. Often everyday activities are affected such as moving in bed, walking, driving, breast feeding (due to discomfort and the effect of analgesia in breast milk), continence (due to associated pelvic floor dysfunction) and safely caring for the child and siblings, especially as the child becomes more mobile.[5] The emotional impacts of "living with enduring pain", include anger and frustration and feelings of "being a burden",[6] with related abuse of analgesics highlighted.[7, 8] Work absenteeism contributes significantly to the economic consequences.

Despite such wide-ranging impacts, symptoms can be overlooked or dismissed by health professionals believing the pain will naturally resolve.[2, 9] However, severe long-lasting pain is often recalcitrant to usual management,[10, 11] and can ultimately require invasive and expensive fluoroscopy guided injections and surgical procedures (ablation and fusion). There is an urgent need for effective strategies to address this personal and societal burden. In particular, guidance is needed for the extended post-partum phase since all current guidance relates to pain during pregnancy and up to 8 weeks post-partum.[12] In light of this, National Health Service (NHS) England has committed to improving access to postnatal physiotherapy.[13]

Pelvic orthoses are one option to manage this condition having support from European guidelines,[14] and the Royal College of Obstetricians and Gynaecologists.[15] These are externally worn devices designed to increase pelvic joint stability, musculoskeletal alignment and sensory input to optimise muscle control, pain and function.[16] There is currently a wide range of "off the shelf" pelvic orthoses available, however they have been shown to be of mixed benefit and often ineffective when pain is severe.[17] Furthermore, women report wear-time issues due to discomfort, lack of ease of use, reduced aesthetics, and impact on movement.[8] Sufficient wear time is however crucial for orthotics as benefits gained are dependent on this.

A novel customised Dynamic Elastomeric Fabric Orthosis (DEFO) for people with PGP has been designed, (DM Orthotics' Ltd, https://www.dmorthotics.com) to address these issues. This pelvic orthosis differs markedly in the design, material and compression grades to "off the shelf" pelvic orthoses. Their use has been previously assessed; during pregnancy, through a randomised controlled trial (RCT), finding that they significantly improved pain over and above the traditional rigid belt;[18] and in a replicated case series of group of eight women experiencing post-partum PGP, identifying an improvement in pain, function and quality of life.[19] Both studies indicated the pelvic DEFOs were acceptable to wear with regard to wear-time, comfort, and aesthetics. These studies together indicate a potentially effective new treatment option to managing this difficult to treat condition in the post-partum period. Further evidence is required to establish the clinical and cost effectiveness of this novel intervention in women experiencing persistent severe PGP following pregnancy. Before considering a definitive trial, the feasibility of running such a trial needs to be tested.

For peer review only - http://bmjopen.bmj.com/site/about/guidelines.xhtml

Aims and objectives

The primary aim is to assess the feasibility and acceptability of the trial procedures comparing the delivery of a novel pelvic orthosis plus standard care with standard care alone in women experiencing severe, chronic PGP post-partum.

The trial objectives are to gain operational experience and gather high quality data to inform the conduct and design of an anticipated definitive RCT and cost-effectiveness analysis, including the most appropriate primary and secondary outcome measures, the most effective recruitment methods, and to inform the sample size calculation of the future trial.

METHODS

Trial design

The EMaPP trial is an assessor blinded, pragmatic, randomised controlled feasibility trial comparing standard care (standardised advice and exercises) to standard care in addition to a customised pelvic orthosis, with an embedded qualitative study. A CONSORT study flowchart outlines the participant pathway through the trial (Figure 1).

Study settings

The three study sites are based in three geographical regions of England: Devon, West Yorkshire, and Buckinghamshire. The interventions are delivered in primary or secondary care subject to local practices.

The trial undertakes a remote, distance-based approach to its delivery at all stages.

- Telephone and virtual screening.
- Interventions delivered via videoconferencing by NHS physiotherapy services using locally approved software.

To prevent digital exclusion, face-to-face intervention will be delivered at a local health care establishment when required.

For peer review only

Eligibility criteria

The study population will comprise women with post-partum PGP, based on the eligibility criteria outlined in Table 1.

Table 1 EMaPP study inclusion and exclusion criteria

Inclusion criteria	Exclusion criteria
Aged ≥ 18 years	Known allergy to Lycra
Able and willing to provide informed consent	Currently pregnant
Self-report persistent PGP (for a minimum of three months post-partum)	Currently wearing a catheter
Self-reported severe PGP (causing walking or stair climbing to be bothersome)	Self-reported history of pathologies causative of lumbo-pelvic pain (e.g. infection, trauma, cancer)
Diagnosis of PGP in line with European guidelines; defined as pain between the posterior iliac crest and inferior gluteal fold, particularly in the sacroiliac joint vicinity, that may radiate to the posterior thigh and occur in conjunction with or separately in the symphysis pubis,[14] captured using the pain referral map.	Participating in concurrent interventional research which may over-burden the patient or confound data collection
Scoring positively with at least one anterior PGP test and two tests for posterior PGP or two positive tests for posterior PGP.[39] [Figure 2]	Participants unable to understand verbal and/or written English adequate to complete the trial procedures/self-report questionnaires. Assistance from a friend/family member for translation purposes is acceptable.
The PGP must have started or been aggravated during pregnancy, as determined by self-report.	

Participant recruitment, identification and screening

A multi-faceted recruitment approach will be undertaken as follows:

Nationally, social media will be used to promote the trial via a various sites including: Mumsnet, Facebook, and Twitter pages of the Pelvic, Obstetric and Gynaecological physiotherapy special interest group, National Maternity Voices, National Childbirth Trusts, and the dedicated study website (https://www.plymouth.ac.uk/research/emapp-trial).

Locally, recruitment will occur via: the Clinical Research Networks who will undertake selected GP database searches; caseloads of physiotherapists working within musculoskeletal, women's health services of the recruiting sites; posters in relevant outpatient clinics/ waiting areas of participating sites; local patient and public involvement and engagement (PPIE) groups and hospital social media accounts.

For peer review only - http://bmjopen.bmj.com/site/about/guidelines.xhtml

The trial will use a dedicated email address, included on all promotional material along with a QR code, to direct interested parties to further information on the trial website. The trial website contains a downloadable participant information sheet (PIS) and reply slip.

All interested participants, and those on databases or caseloads who are deemed potentially eligible, will be sent a trial information pack containing: PIS; details of the ethically approved study sites; and a reply slip to indicate their interest and confirm they feel they are eligible.

Eligibility screening process

Eligibility screening will occur via a two-stage process. On receipt of the completed reply form a telephone screen (stage 1) will be undertaken using a pre-formatted screening checklist based on the eligibility criteria. If deemed eligible, a video conference physical screening assessment will be arranged (stage 2). A screening pack will be sent out ahead of this physical screening appointment containing: the screening battery tests (schema of physical tests and instructions), Beighton self-report score, trial consent forms, orthotic measuring guide and tape measure (to capture measurements for production of the customised orthosis, should the participant be allocated to the intervention group) and digital callipers.

The physical screening requires the presence of a partner/trusted other to assist with the physical assessment and orthosis measurements, under the guidance of the remote researcher. All screen failures will be recorded.

Randomisation

Once eligibility is confirmed, the consent process completed, and screening data entered into the trial database, participants will be sent a text/email, generated by the Peninsula Clinical Trials Unit (PenCTU), requesting them to complete the baseline questionnaires. Once complete, the participant will be randomly allocated on a 1:1 ratio, using random permuted blocks, stratified by centre and presence/absence of lumbo-pelvic pain pre-pregnancy, to either the standard care or intervention group (figure 1). Screening data capture and randomisation is achieved via a web-based system created by the PenCTU.

After randomisation, a series of emails will be automatically generated. A blinded email will be sent to the research team members involved in eligibility screening. An un-blinded email will be sent to the Chief Investigator (CI), study administrator and site Principal Investigator (PI). An email/text will be sent to the participant to inform them of their group allocation.

Blinding

The trial participants and NHS treating physiotherapists are unable to be blinded due to the nature of the intervention. Every effort will be made to ensure the research team is blinded to group allocation throughout the trial. The statisticians will remain blind to group allocation when conducting the primary analyses until analyses that necessitate un-blinding need to be performed, such as wear-time analysis. The research team will keep a detailed log of any inadvertent un-blinding events. Final un-blinding of the research team will occur after database lock and blinded analysis has been undertaken.

For peer review only - http://bmjopen.bmj.com/site/about/guidelines.xhtml

Interventions

NHS physiotherapists will provide all interventions at the two protocolised video-based sessions. At the participant's preference, face to face sessions can be undertaken in place of these remotely delivered sessions. The initial session will last a maximum of 60 minutes and the follow-up session (a maximum of 30 minutes) will be scheduled approximately 10 days later. Participants are not prevented from receiving other care. This will be captured in the health resource use questionnaire.

Standard care group (standardised information and exercise)

The standard care regime was designed in collaboration with women's health physiotherapists experienced in the treatment of persistent PGP. This was considered by them to be reflective of usual clinical practice. It comprises two components:

Standardised information:

This will be provided through a discussion centred on the publicly available booklet produced by the Pelvic, Obstetric and Gynaecological Physiotherapy special interest group "Guidance for mothers-to-be and New Mothers: Pregnancy-related Pelvic Girdle pain" (https://pogp.csp.org.uk/system/files/pogp-pgppat_3.pdf).

Standardised exercise:

The physiotherapist will teach participants a programme of up to four lumbo-pelvic/hip exercises, typical of those provided within usual physiotherapy practice. These will be selected from a protocolised set (supplementary file 1), on the basis of their initial assessment. Participants will be advised to complete the exercises on three days of the week, with each session lasting approximately 20-30 minutes. All participants will receive an exercise leaflet describing the exercises, with accompanying images. This will be posted or emailed dependent on participant preference.

Intervention group (standardised information and exercise plus DEFO)

In addition to standard care, participants allocated to the intervention group will be provided with two pairs of customised DEFOs (figure 3, DM Orthotics Ltd, https://www.dmorthotics.com). The orthosis will be worn for the first time at the initial intervention session, enabling the physiotherapist to check the fit; this will also be reviewed at the follow up session. Participants will be advised to wear the DEFO for a maximum of 12 hours per day, with wear time graduated over the course of the first five days in line with manufacturer guidance. The orthoses are delivered with an advice booklet detailing donning and doffing, wear time and care instructions, with a supplementary video hosted on the trial web page. Where alterations are required because of poor fit, in line with current practice the NHS physiotherapists will follow a standard operating procedure, making contact with the orthotic company to arrange an adjustment if required.

For peer review only - http://bmjopen.bmj.com/site/about/guidelines.xhtml

Data collection

Feasibility outcomes

The trial outcomes include feasibility and acceptability of the EMaPP trial. Quantitative and qualitative feedback will be obtained to identify the main determinants of experience and acceptance of the trial in line with the trial objectives. In particular, the following measures and operational criteria will be determined:

- Number and proportion of eligible participants from the target population.
- Willingness of participants to be randomised.
- Recruitment and retention rates of eligible participants through the trial.
- Completeness of data sets/outcome measures.
- Incidence of face to face sessions.

Clinical outcome measures

A battery of standardised, patient reported outcome measures, together with demographic and diagnostic characteristics (birth details, pain history, Beighton Score,[20, 21] and Two Point Estimation (2PE) Task,[22,23] will be collected at baseline. A selection of outcome measures will be repeated fortnightly and at 12 and 24-weeks after the first intervention session to map in more detail the trajectory of pain over the trial timeline (table 2). All patient reported outcome measures will be reported directly by the participants via the web-based app. At each time point, participants will automatically receive five text/email reminders (one per day) if the data entry has not started. All patient reported outcome measures included within this study align with the PGP core outcome set for evaluating the effectiveness of interventions in post-partum PGP.[24]

For peer review only

For peer review only - http://bmjopen.bmj.com/site/about/guidelines.xhtml

Table 2: Assessment schedule

**Assessments**	Baseline	Post-initial appointment		
		Fortnightly	12 weeks	24 weeks
Demographics, birth details, pain history	x			
Pain medication use	x	x	x	x
Numerical Pain Rating Scale [25, 26]	x	x	x	x
Pelvic Girdle Questionnaire [40] to assess function and symptoms	x		x	x
Short-form 36 [41] to assess health-related quality of life and calculate quality adjusted life-years (QALYS)	x		x	x
EQ-5D-5L to assess health-related quality of life and calculate QALYS [42]	x		x	x
Edinburgh Post-natal Depression Scale [33] to assess post-natal depression (cut-off ≥ 12 points)	x		x	x
International Consultation on Incontinence Questionnaire, Urinary Incontinence - Short form to assess level and impact of symptoms of incontinence [43]	x		x	x
Freemantle Back Awareness Questionnaire **to** assess explicit body perception of the lumbo-pelvic region[36,37]	x		x	x
Tampa Scale Kinesiophobia to assess fear of movement/kinesiophobia[44]	x		x	x
Health and Social Care Resource use Questionnaire to collect data on health, social and wider care resource use[18]	x		x	x
Beighton Score[20, 21] to determine joint hypermobility	x			
Two-point estimation task[22, 23] to determine implicit somatoperception	x			x
Wear time adherence (Orthotimer)				x
Qualitative interviews				x

For peer review only - http://bmjopen.bmj.com/site/about/guidelines.xhtml

Proposed clinical outcome measures

Numerical Rating of Pain Scale (NRPS) is the proposed primary outcome measure for the definitive RCT. This 0-10 point scale is widely used,[25] quick to complete, and with a format suitable for completion through a mobile/app. There is evidence in chronic pain patients to suggest a 1-point change is clinically significant,[26] and hence a difference between groups of >1.0 at 24-week follow up will indicate a signal of efficacy. Participants will rate their pain experience over the past fortnight using the NPRS within four categories: i) worst level of day-time pelvic pain, ii) average level of day-time pelvic pain, iii) worst level of night-time pelvic pain, iv) average level of night-time pelvic pain.

The proposed secondary outcome measures are detailed in Table 2.

Resource use and costs associated with delivery of both intervention arms will be estimated. Resource use associated with the measurement, ordering, delivery and production of the orthosis, and the physiotherapy interventions, will be collected via within trial reporting, including participant level contact and non-contact time for NHS staffing input.

Intervention fidelity

NHS clinicians will complete a pre-formatted intervention fidelity checklist for each of the two physiotherapy sessions. Adherence to wearing the orthosis will be assessed via a temperature sensor integrated into the seams of the orthosis. The Orthotimer sensor (Rollerwerk Medical Engineering, Balingen, Germany) is embedded in a small (13mm × 9mm × 5 mm), dust and watertight unit. It will record time, date, and temperature every 15 minutes over the 24-week period with temperature precision of ± 0.1°C. DM Orthotics Ltd will activate and sew the sensor into each orthosis before dispatching to participants. Adherence to the home exercise programme is not assessed during the trial, however it is explored via the qualitative interviews.

Adverse events

Throughout the trial, all possible precautions will be taken to ensure participant safety and wellbeing. Participants will be monitored for adverse events throughout the 24-weeks. The likelihood of participants being harmed by either wearing the orthosis or undertaking the exercise programme (which reflects usual clinical practice) or any of the trial procedures is considered very low. Collection and reporting of adverse events will be captured via the web-based app and relate to those classified as an adverse reaction (including adverse device effects (ADEs)) or those which are serious. These will be collected at fortnightly intervals. Upon receipt of a reported reaction or serious adverse event, the CI will contact the local PI to discuss causality. All adverse events will be reported at the Trial Steering Committee (TSC) and Trial Management Group (TMG) meetings.

Qualitative sub-study

The aim is to explore the experiences of wearing (participant) and providing (clinicians) the pelvic orthosis and engaging in the trial procedures.

The specific objectives are to investigate acceptability of the trial methods across both arms, acceptability (comfort, wear time) of the orthosis, impact the intervention may/may not have on the participant's life, and adherence to the exercise regime.

For peer review only - http://bmjopen.bmj.com/site/about/guidelines.xhtml

Semi-structured telephone interviews will be undertaken, at the end of the trial, with 10 purposively sampled participants (n= 5 intervention group, n= 5 standard care group) and five NHS physiotherapists involved in delivering the interventions. A topic guide will be used (Supplementary file 2). Interviews will be digitally recorded and transcribed verbatim.

Informal telephone exit interviews will be undertaken with participants that withdraw from the trial to share their reason for withdrawing. The interviews will not be recorded or transcribed. Field notes will be taken.

End of trial definition

There are no formal stopping criteria. It will be prematurely stopped on safety grounds through a decision by the TMG and TSC.

Sample size

As this is a feasibility trial, a formal sample size calculation, based on power for detecting a clinically meaningful between-group difference in a primary clinical outcome, will not be undertaken. The trial aims to recruit 60 participants over a seven-month period. The sample size is pragmatic and determined to be large enough to provide robust estimates on the likely recruitment rates and follow up and estimates of the variability on the proposed primary outcome to inform a future sample size calculation. The target is set to enable determination of whether it would be practicable to recruit adequate numbers in a manner conducive to implementing a reasonably costed definitive trial. A sample size of 60 will allow overall retention rate at six-month follow-up to be estimated to within a 95% confidence interval of approximately ±13% (±10% if retention rate is 80%). Assuming a non-differential retention rate of 80%, this should provide follow-up outcome data on a minimum of 24 participants per group.

Patient and public involvement and engagement (PPIE)

Women with experience of severe chronic PGP provided input into the study design. Priority of the research question, choice of outcome measures, recruitment methods, and mode of intervention delivery were informed by discussions with women through a focus group session and ongoing discussions with a dedicated lay member of the project team. Lay input continues through membership on the TMG, and an additional independent lay member on the TSC. PPIE will play a key role in the dissemination of trial findings, contributing to and reviewing material.

Determining progression to the definitive trial

Progression to a full trial application will occur if minimum success criteria are achieved in key feasibility aims and objectives, and/or if solutions can be identified to overcome any issues. These criteria will be finalised in discussion with the TSC. A red, amber, green system will assist the decision-making progress, with Red indicating “Stop”, Amber indicating “Discuss and Modify” and Green indicating “Go”. The anticipated progression criteria are detailed in table 3.

For peer review only - http://bmjopen.bmj.com/site/about/guidelines.xhtml

Table 3: Progression criteria	
Criteria	RAG rating
60 participants recruited within a 7-month recruitment window	Red: <60%<br>Amber: 60-80%<br>Green: > 80%
Percentage of participants randomised to intervention group non-compliant in wearing the shorts (non-compliance – wearing the shorts for less than 6 hours/day or total of 42 hours/week)	Red: > 70%<br>Amber: 50-70%<br>Green: <50%
Percentage of participants completing primary outcome measure (NRPS) at 24-week follow up	Red: < 60%<br>Amber: 60-80%<br>Green: > 80%
* Percentage of participants completing secondary outcome measures at 24 week follow-up in (in the following order of priority (EQ-5D-5L, SF-36, PGQ, ICIQ)	Red < 60%,<br>Amber 60-80%,<br>Green > 80%
Evidence to suggest efficacy, i.e. that the support shorts hold promise as an effective intervention, demonstrated by an 80% confidence interval that indicates plausibility of the between group difference in the primary outcome measure being ≥ 1 point, on the NPRS.	N/A
Total resource estimated to conduct the definitive trial within levels likely to attract funding.	N/A

RAG rating = Red Amber Green Rating

Table 3: Progression criteria. *This progression criteria relates to the selection of outcome measures to be used in a definitive trial. It does not influence the decision as to whether or not there should be progression to a definitive trial.

ETHICS AND DISSEMINATION

The EMaPP trial has received approval from the: Research Ethics Committee (REC) (REC reference number: 21/WM/0155); Health Research Authority (HRA); and confirmation of capacity and capability from the Research and Development departments of the participating centres. Any amendments to the protocol will be discussed with the sponsor and submitted to HRA/REC for approval.

Dissemination will target users, clinicians and researchers. The results will inform the design of the anticipated definitive trial. It will not inform clinical decision making since clinical and cost effectiveness cannot be determined at this level. Dissemination, regardless of outcome will be via: publication of results in a peer reviewed journal, a funding proposal for a full scale effectiveness trial (if progression criteria are met), lay orientated research feedback events and a lay summary for trial participants, staff and the general public, and via conference presentations at grass roots physiotherapy events and research conferences. The study report will be accessible on the study web page (https://www.plymouth.ac.uk/research/emapp-trial), as will the trial protocol and statistical analysis plan. The protocol has been written and published in line with SPIRIT guidelines.[27] Publications will follow CONSORT guidance for feasibility trials,[28] and the template for intervention description and replication (TiDIER) guidelines.[29] Authorship of the intended articles will be the study team; professional writers will not be used.

For peer review only - http://bmjopen.bmj.com/site/about/guidelines.xhtml

Economic evaluation

The resources required to provide the intervention will be assessed and a framework established for a future cost-effectiveness analysis alongside a definitive RCT. Data on intervention resources will be collected via within-trial reporting, including participant-level contact and non-contact time, and training for delivery staff. Participants will self-report health, social and wider care resource use, using the Resource Use Questionnaire. Participants will complete the EQ-5D-5L (the anticipated primary economic outcome measure in a full trial), and QALYs will be estimated over the follow-up period. The appropriateness of the SF-6D (based on the SF-36) for estimating QALYs will be explored. The economic evaluation methods will be developed to provide a future policy-relevant cost-effectiveness analysis of the intervention in the context of the UK NHS/Personal Social Services.

Data management and analysis

Detailed data management activities are described in a separate Data Management Plan. PenCTU staff will monitor completeness and quality of data throughout the trial. No imputation of missing data will be undertaken.

Quantitative

A detailed statistical analysis plan (SAP) will be finalised ahead of trial database locking. A CONSORT diagram will display data from screening, recruitment, consent and follow up logs and be used to generate estimates of eligibility, recruitment, consent and follow-up rates. Completion rates will be estimated for outcome measures at each time-point, including the health, social and wider care resource-use data. Recruitment and retention rates will be accompanied by 95% confidence intervals, to inform assumptions for planning the definitive trial. Adherence data (wear-time) will contribute to evaluation of intervention acceptability/feasibility and evaluated as total hours and as a percentage of total possible daytime wearing time (12 hours/day). The percentage of week's participants wore the shorts for a minimum of 42 hours a week, and the percentage of days participants wore the shorts for a minimum of 6 hours a day, will be summarised.

All outcomes will be summarised by allocated group at each follow-up with appropriate descriptive statistics. Between-group differences will be reported with 80% confidence intervals in addition to 95% confidence intervals, with particular focus on change in pelvic girdle day-time pain at the primary end point of 24 weeks. According to the 2008 IMMPACT consensus statement,[26] changes of one point represent minimally important decreases in pain using the NRPS of pain intensity. We will inspect the confidence interval of the between group difference to see if there is evidence of a plausible signal of efficacy. Estimates of the variability of outcome measures and the correlation between baseline and follow-up outcome measures will inform the sample size calculation for a future definitive RCT.

Qualitative

Thematic analysis will be used for the telephone interview data using NVivo 12 software for organisation and analysis (QSR International, Southport, UK, July 2021). Braun and Clarke's six-phase process will be used: (i) data familiarisation; (ii) coding; (iii) generating initial themes; (iv) reviewing themes; (v) defining and naming themes and (vi) writing up to identify patterns of meaning within

For peer review only - http://bmjopen.bmj.com/site/about/guidelines.xhtml

the data sources. Initial themes will be refined by two researchers to maximise credibility and dependability.

Interviewees will be invited to review a draft of the analysis to ensure accurate representation of their views/experiences.

Discussion

This is the first RCT to explore the feasibility and acceptability of a novel customised pelvic orthosis in the management of chronic PGP in the post-partum period. There is a lack of evidence to support the management of this condition beyond the first eight weeks postpartum. This orthosis has already shown promise as an intervention during pregnancy,[18] and in a replicated case series study of eight women post-partum.[19] This trial aims to serve as a starting point to investigate the effectiveness of this orthosis in the management severe chronic PGP post-partum.

Some aspects of the study design are noteworthy. All trial procedures and intervention delivery are undertaken remotely; described as a virtual trial.[30, 31] This replicates current practice, mitigates the risk associated with face-to-face appointments during the COVID-19 pandemic, and ensures trial delivery in the event of a lockdown. It is also a potentially more efficient approach.[31] Because some participants may not be able to participate in a fully virtual trial, to optimise generalisability provisions have been made to provide a non-virtual format when this is preferred by the participant. Monitoring of the proportion of women who choose a non-virtual approach will further inform the future trial design.

All outcome measures collected at follow up time points (apart from adherence to wear time in the intervention group) are patient reported outcome measures (PROMs), and via a web-based app. This approach was chosen on the basis of PPIE input, the rationale being to minimise participant burden. The collection of PROMs minimises the influence of the research team on data collection. Recommendations from Mercieca-Bebber,[32] have been used to improve PROMS data collection during this trial, such as reducing free text options in questionnaires, flexible data entry, and text/email reminders to enhance data completeness. With regard to wear time adherence data, this too is collected remotely using the orthotimer temperature sensor; with the added advantage of providing detailed and objective data which is otherwise challenging to reliably collect via methods such as patient diaries.

The qualitative interviews are also undertaken via remote means. This decision was informed by PPIE discussions, aiming to reduce participant burden in terms of travel time and inconvenience; particularly important considerations for people in pain and looking after young children.

The self-report depression EPDS questionnaire[33] is used to assess post-natal depression. If a score of > 12 points (indicative of depression) is recorded, an email to the research team is triggered to contact the participant and their GP, thereby to ensuring duty of care is upheld. The data collected will have the added advantage of increasing understanding of the prevalence of post-natal depression in this population; currently an under-researched area.

Persistent PGP is difficult to manage with the aetiology of the condition,[34, 35] and the mechanism of action of pelvic orthotics under debate. One factor that has recently been under the spotlight within this group and may represent a possible mechanism of action for an orthotic, relates to changes in somatoperception (the perception of size and shape), with evidence pointing to an

For peer review only - http://bmjopen.bmj.com/site/about/guidelines.xhtml

altered explicit somatoperception in both the peri-natal,[36] and post-natal period.[37] This study has included explicit (via the FreBAQ questionnaire) and implicit (measure of two-point estimation) measures of somatoperception, as recommended by Viceconti.[38] It is the first to study both types of somatoperception in parallel during an interventional trial.

Availability of data and material

The authors declare the data will be made available to the scientific community with as few restrictions as feasible, while retaining exclusive use until the publication of major outputs.

Author's Contributions: All authors contributed to the conception and design of the trial. BH drafted the manuscript and all co-authors provided critical revisions to the clinical and intellectual content. All authors have read and approved the manuscript.

Abbreviations: CI: Chief Investigator; CRN: Clinical Research Network; CTU: Clinical Trial Unit: DEFO: Dynamic Elastomeric Fabric Orthosis; EPDS: Edinburgh Post-natal Depression Scale: FreBAQ: Freemantle Back Awareness Questionnaires; GP: General Practitioner; ICIQ UI –SF: International Consultation Incontinence Questionnaire Urinary Incontinence - Short Form; NHS: National Health service; NRPS: Numerical Rating of Pain Scale; PGP: Pelvic Girdle Pain; PGOP: Pelvic Gynaecological and Obstetric Physiotherapists; PPIE: Patient and Public Involvement and Engagement; PGQ: Pelvic Girdle Questionnaire; PIS: Participant Information sheet; QALY: Quality Adjusted Life Year; TMG: Trial Management Group; TSC: Trial Steering Committee; TSK: Tampa Scale Kinesiophobia

Competing interests: The research team have no competing interests.

Administrative information

Protocol version: Version 3

Funding: This study is funded by the NIHR Research for Patient Benefit Programme (NIHR201930), United Kingdom. This article presents independent research. The views expressed are those of the authors and not necessarily those of the NHS, the NIHR or the Department of Health.

Name and contact information for the trial Sponsor: Royal Cornwall Hospitals NHS trust (RCHT), Research and Development Department, B18, The Knowledge Spa, Truro, Cornwall, TR1 3LJ

Role of the sponsor and funder: The sponsor RCHT assumes overall responsibility for the initiation and management of the trial. The sponsor and funder will not have direct involvement in trial design, conduct, data analysis and interpretation, manuscript writing, and dissemination of results.

Trial Steering Committee: This committee has an independent chair, an independent physiotherapist, midwife, statistician and patient representative. It meets every six months to review the progress of the trial and any serious adverse events and will report to the sponsor. DMC is not required in this trial given the low-risk interventions.

Trial Management Group: Chaired by the Chief Investigator (Prof Freeman) and includes a representative from the sponsor, PenCTU, trial statistician, health economist and patient representative. It also has representation from co-investigators and leads for the qualitative and

For peer review only - http://bmjopen.bmj.com/site/about/guidelines.xhtml

health economic components. The TMG will meet monthly to review trial progress and ensure appropriate management of the trial.

Supplementary file 1: Standardised exercise regime

Supplementary file 2: Topic guide for qualitative interviews

Figure Legends

Figure 1 – Trial flow chart

Figure 2 – Pelvic girdle pain screening battery

Figure 3 – Customised pelvic support shorts (DEFO)

For peer review only

For peer review only - http://bmjopen.bmj.com/site/about/guidelines.xhtml

REFERENCE LIST

1) Kovacs F, Garcia E, Royuela A, et al. Prevalence and factors associated with Low back pain and pelvic girdle pain during pregnancy: a multicentre study conducted in the Spanish national health service. *Spine* 2012;37:1516-1533 doi: 10.1097/BRS.0b013e31824dcb74
2) Wuytack F, Curtis E, Begley, C. Experiences of First-Time Mothers with Persistent Pelvic Girdle Pain after Childbirth: Descriptive Qualitative Study. *Physical Therapy 2015;*95**:** 1354-1364 doi: 10.2522/ptj.20150088
3) Bergström C, Persson M, Nergård K, et al. Prevalence and predictors of persistent pelvic girdle pain 12 years postpartum. *BMC Musculoskeletal Disorders* 2017*;*18**:**399 doi: 10.1186/s12891-017-1760-5
4) Wuytack F, Daly D, Curtis E, et al. Prognostic factors for pregnancy-related pelvic girdle pain, a systematic review. *Midwifery* 2018;66**:**70-78 doi: 10.1016/j.midw.2018.07.012
5) Mackenzie J, Murray E, Lusher J. Women's experiences of pregnancy related pelvic girdle pain: A systematic review. *Midwifery* 2018;56:102-111 doi: 10.1016/j.midw.2017.10.011
6) Persson M, Winkvist A, Dahlgren L, et al. "Struggling with daily life and enduring pain": a qualitative study of the experiences of pregnant women living with pelvic girdle pain. *BMC Pregnancy and Childbirth* 2013;13**:** 111 doi: 10.1186/1471-2393-13-111
7) Bergström C, Persson M, Mogren I. Sick leave and healthcare utilisation in women reporting pregnancy related low back pain and/or pelvic girdle pain at 14 months postpartum. *Chirop Man Therap* 2016;24:7 doi: 10.1186/s12998-016-0088-9
8) Malmqvist S, Kjaermann I, Andersen K, et al. Can a bothersome course of pelvic pain from mid-pregnancy to birth be predicted? A Norwegian prospective longitudinal SMS-Track study. *BMJ Open;* 2018:8 doi: 10.1136/bmjopen-2017-021378
9) Close C, Sinclair M, Liddle D. Women's experience of low back and/or pelvic pain (LBPP) during pregnancy. *Midwifery,* 2016; 37**:**1-8 doi: 10.1016/j.midw.2016.03.013
10) Szkwara J, Milne N, Hing W, et al. Effectiveness, Feasibility, and Acceptability of Dynamic Elastomeric Fabric Orthoses (DEFO) for Managing Pain, Functional Capacity, and Quality of Life during Prenatal and Postnatal Care: A Systematic Review. *International Journal of Environmental Research and Public Health,* 2019; 16**:** 2408 doi: 10.3390/ijerph16132408
11) Richards E, Van Kessel G, Virgara R, et al. Does antenatal physical therapy for pregnant women with low back pain or pelvic pain improve functional outcomes? A systematic review. *Acta Obstetricia et Gynecologica Scandinavica* 2012;91**:**1038-1045 doi: 10.1111/j.1600-0412.2012.01462.x
12) National Institute Clinical Excellence (NICE). Postnatal care: Clinical Guideline: 2021. https://www.nice.org.uk/guidance/ng194/resources/postnatal-care-pdf-66142082148037 Accessed: Jan 2022
13) Health Education England. The engagement of allied health professionals and psychologists in the maternity care pathway: 2019. https://www.hee.nhs.uk/ourwork/allied-health-professions/working-maximise-contribution-allied-health-professions Accessed: September 2021
14) Vleeming A, Albert H, Ostgaard, H, et al. European guidelines for the diagnosis and treatment of pelvic girdle pain. *Eur Spine J* 2008;17**:**794-819 doi: 10.1007/s00586-008-0602-4
15) Royal College of Obstetricians and Gynaecologists. https://www.nhs.uk/conditions/pregnancy-and-baby/pelvic-pain-pregnant-spd/. Accessed September 2020
16) Mens J, Pool-Goudzwaard, A. The transverse abdominal muscle is excessively active during active straight leg raising in pregnancy-related posterior pelvic girdle pain: an observational study. *BMC Musculoskelet Disord* 2017;18:372 doi: 0.1186/s12891-017-1732-9
17) Ho S, Yu W, Lao T, et al. Comfort evaluation of maternity support garments in a wear trial. *Ergonomics 2008;*51**:** 1376-1393 doi: 10.1080/00140130802116489

For peer review only - http://bmjopen.bmj.com/site/about/guidelines.xhtml

18) Cameron L, Marsden J, Watkins K, et al. Management of antenatal pelvic girdle pain study (MAPS): A double blinded randomised trial evaluating the effectiveness of two pelvic orthoses. *Int. J. Women's Health Care* 2018;3**:**1-9.
19) Cameron L. Management of Chronic Post Partum Pelvic Girdle Pain: MOPS. https://clinicaltrials.gov/ct2/show/NCT02648607
20) Cooper D, Scammell B, Batt M, et al Development and validation of self-reported line drawings of the modified Beighton score for the assessment of generalised joint hypermobility. *BMC Medical Research Methodology* 2018;18:11 doi: 10.1186/s12874-017-0464-8
21) Beighton P, Solomon L, Soskolne C. Articular mobility in an African population. *Ann Rheum Dis* 1973:32:5 413-418 doi: 10.1136/ard.32.5.413
22) Adamczyk W, Luedtke K, Saulicz O, et al. Sensory dissociation in chronic low back pain: Two case reports. *Physiotherapy Theory and Practice***,** 2018;34:8 1-9 doi: 10.1080/09593985.2017.1423431
23) Adamczyk W, Sługocka A, Mehlich K, et al. Preliminary Validation of a Two-Point Estimation Task for the Measurement of Sensory Dissociation in Patients with Chronic Low Back Pain. *Pain Medicine* 2019;20**:**2472-2478 doi: 10.1093/pm/pny220
24) Remus A, Smith V, Gutke A, et al. A core outcome set for research and clinical practice in women with pelvic girdle pain: PGP-COS. *PLOS ONE,* 2021;16:2 doi: 10.1371/journal.pone.0247466
25) Childs J, Piva S, Fritz J. Responsiveness of the numeric pain rating scale in patients with low back pain. *Spine 2005 (Phila Pa 1976);*30**:**1331-4 doi: 10.1097/01.brs.0000164099.92112.29
26) Dworkin R, Turk D, Wyrwich K, et al. Interpreting the clinical importance of treatment outcomes in chronic pain clinical trials: IMMPACT recommendations. *Journal of Pain,* 2008 9**,** 105-21 doi: 10.1016/j.jpain.2007.09.005
27) Chan A, Tetzlaff J, Altman D, et al. SPIRIT 2013 statement: defining standard protocol items for clinical trials. *Ann Intern* Med 2013;38:(6) 506–14 doi: 10.7326/0003-4819-158-3-201302050-00583
28) Eldridge S, Chan C, Campbell M, et al. CONSORT 2010 statement: extension to randomised pilot and feasibility trials. *BMJ* 2016; 355;i5239 doi: 10.1136/bmj.i5239
29) Hoffmann T, Glasziou P, Boutron I, et al. Better reporting of interventions: template for intervention description and replication (TIDieR) checklist and guide. *BMJ* 2014 348**,** g1687-g1687 doi: 10.1136/bmj.g1687
30) National Academies of Sciences, E., Medicine, Health, Medicine, D., Board on health sciences, P., Forum on drug discovery, d. & translation 2019. The National Academies Collection: Reports funded by National Institutes of Health. *In:* Shore, C., Khandekar, E. & Alper, J. (eds.) *Virtual Clinical Trials: Challenges and Opportunities: Proceedings of a Workshop.* Washington (DC): National Academies Press
31) Dorsey E, Wagner J, Bull M, et al. Feasibility of Virtual Research Visits in Fox Trial Finder. *J Parkinsons Dis* 2015;5**:**505-15 doi:10.3233/JPD-150549
32) Mercieca-bebber R, Palmer M, Brundage M, et al. Design, implementation and reporting strategies to reduce the instance and impact of missing patient-reported outcome (PRO) data: a systematic review. *BMJ Open* 2016: 6, e010938 doi: 10.1136/bmjopen-2015-010938
33) Cox J, Holden J, Sagovsky R. Detection of Postnatal Depression. *British Journal of Psychiatry* 1987;150**:**782-786 doi: 10.1192/bjp.150.6.782
34) Meijer O, Hu H, Wu W, et al. The pelvic girdle pain deadlock: 1. Would 'deconstruction' help? *Musculoskeletal Science &* Practice 2020;48**:** 102169 doi: 10.1016/j.msksp.2020.102169
35) Meijer O, Barbe M, Prins M, et al. The Pelvic Girdle Pain deadlock: 2. Topics that, so far, have remained out of focus. *Musculoskeletal Science &* Practice 2020;48**:** 102166 doi: 10.1016/j.msksp.2020.102166

For peer review only - http://bmjopen.bmj.com/site/about/guidelines.xhtml

36) Wand B, Elliott R, Sawyer A, et al. Disrupted body-image and pregnancy-related lumbopelvic pain. A preliminary investigation. *Musculoskeletal Science and Practice* 2017;30:49-55 doi: 10.1016/j.msksp.2017.05.003
37) Beales D, Lutz A, Thompson J, et al. Disturbed body perception, reduced sleep, and kinesiophobia in subjects with pregnancy-related persistent lumbopelvic pain and moderate levels of disability: An exploratory study. *Manual Therapy* 2016;21:69-75 doi: 10.1016/j.math.2015.04.016
38) Viceconti A, Camerone E, Luzzi D, et al. Explicit and Implicit Own's Body and Space Perception in Painful Musculoskeletal Disorders and Rheumatic Diseases: A Systematic Scoping Review. *Frontiers in Human Neuroscience* 2020;14:83 doi: 10.3389/fnhum.2020.00083
39) Fagevik Olsén M, Körnung P, Kallin S, et al. Validation of self-administered tests for screening for chronic pregnancy-related pelvic girdle pain. *BMC Musculoskeletal Disorders,* 2021;22: 237 doi: 10.1186/s12891-021-04103-0
40) Stuge B, Garratt A, Krogstad Jenssen H, et al. The Pelvic Girdle Questionnaire: A Condition-Specific Instrument for Assessing Activity Limitations and Symptoms in People with Pelvic Girdle Pain. *Physical Therapy* 2011;91:1096-1108 doi: 10.2522/ptj.20100357
41) Wuytack F, O'Donovan M. Outcomes and outcomes measurements used in intervention studies of pelvic girdle pain and lumbopelvic pain: a systematic review. *Chirop Man Therap* 2019;27:62 doi: 10.1186/s12998-019-0279-2
42) Whitehead S, Ali S. Health outcomes in economic evaluation: the QALY and utilities. *British medical bulletin* 2010;*96*:5–21 doi: https://doi.org/10.1093/bmb/ldq033
43) Avery K, Donovan J, Peters T, et al. ICIQ: A brief and robust measure for evaluating the symptoms and impact of urinary incontinence. *Neurourology and Urodynamics* 2004;23: 322-330 doi: 10.1002/nau.20041
44) Miller R, Kori S, Todd D. The Tampa Scale: a Measure of Kinesiophobia. *The Clinical Journal of Pain* 1991;7:51.

For peer review only - http://bmjopen.bmj.com/site/about/guidelines.xhtml

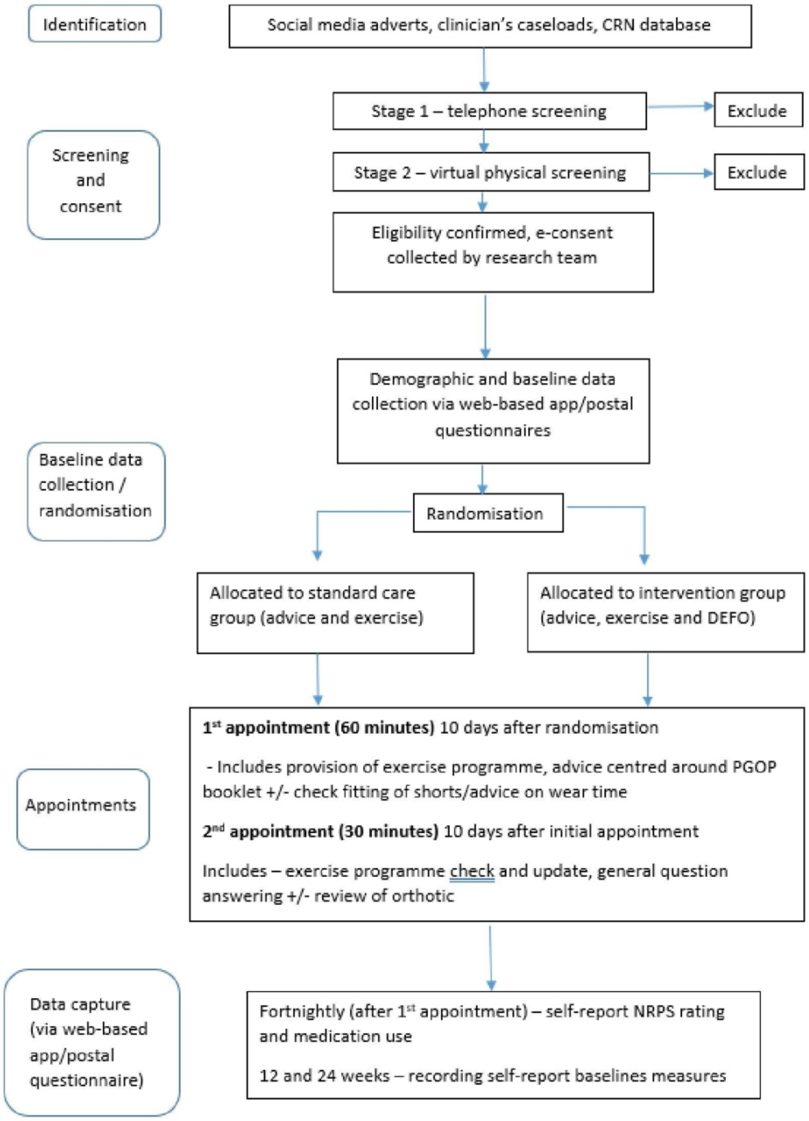

Trial flow chart

159x200mm (300 x 300 DPI)

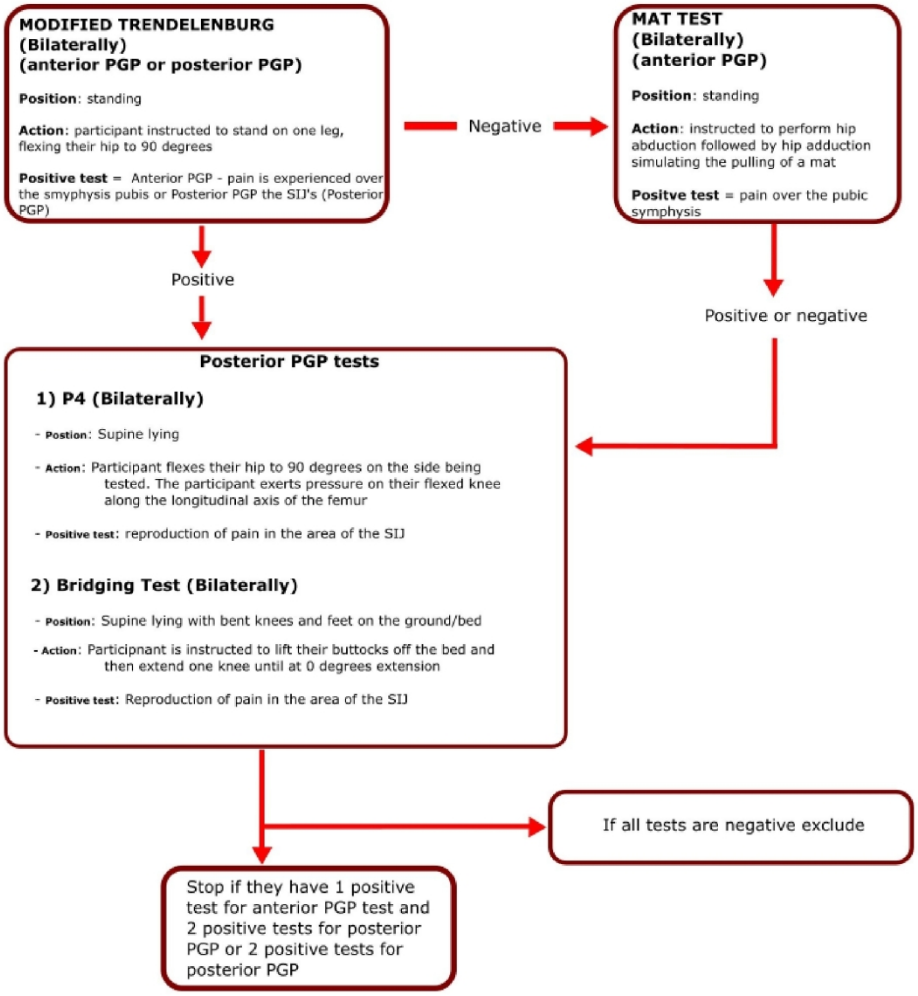

Pelvic girdle pain screening battery

159x200mm (300 x 300 DPI)

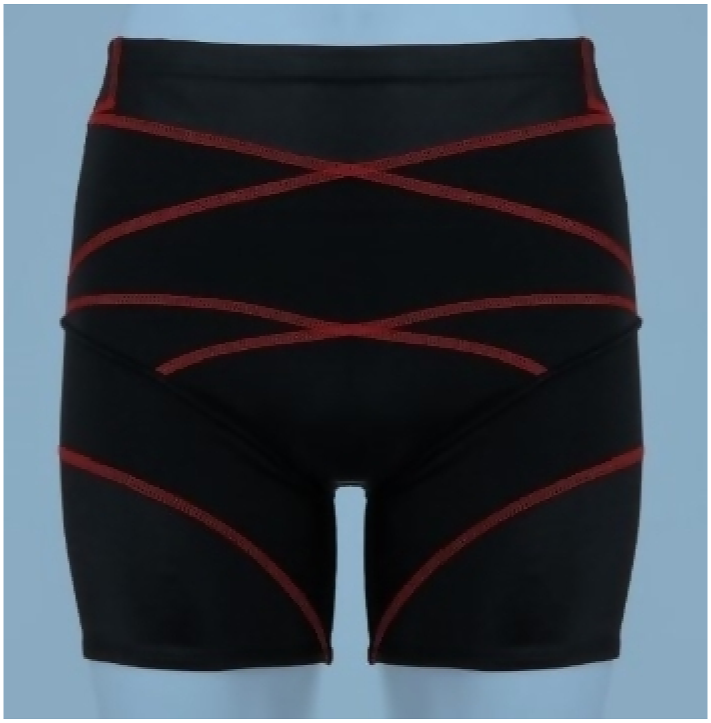

Customised pelvic support shorts (DEFO)

29x30mm (300 x 300 DPI)

Supplementary file 1: Standardised Exercise regime

Reference: Exercises adapted from "Exercise and Advice after pregnancy" PGOP booklet. https://pogp.csp.org.uk/system/files/publication_files/POGP-E%26AA5%28UL%29.pdf

Location: completed at home independently.

Frequency: Aim x 3 per week

Dosage: Aim for up 20 - 30 min's per session. Participants may need to break this into 2 parts.

Repetitions: Exercises described have suggested sets and repetitions. However, this should be guided by treating clinician and the women's capacity to exercise.

Quantity: Choose a maximum of 4 exercises

Advice to be provided to all women: If any of the exercises cause pain then reduce the amount of repetitions that you are performing. If this continues on more than two occasions, then cease the exercise and discuss with your clinician at your follow up appointment.

Permitted exercises are described below:

1) Pelvic floor exercises
   - *Exercise description*: Imagine that you are trying to stop yourself passing urine or wind: 'squeeze and lift' your pelvic floor muscles, closing and drawing up the 3 passages. You may not feel that much is happening at first but keep trying. Hold the squeeze for a few seconds, and then relax for a few seconds. Do not hold your breath. You may find that abdominal hollowing takes place at the same time.
   - *Positions* – consider the use of different position to exercise in (e.g. standing, sitting and lying),
   - *Speed of contraction/Dosage*:
     - *For endurance:* Start gently and rhythmically. Gradually increase the hold time and the number that you do until you can hold the squeeze for up to 10 seconds and repeat up to 15 times in a set (up to 3 sets)
     - *For speed*: It is also important that the pelvic floor muscles are able to react quickly to stop you leaking when you cough, sneeze, laugh or shout. Tighten them as quickly and strongly as you can, and then relax. Do this between 8-15 times a set with up to 3 sets.
   - *Advice*: Always tighten your pelvic floor muscles before and during any activity requiring physical effort; for example, when lifting, coughing or sneezing

2) Abdominal hollowing exercise:
   - *Exercise description*: Place one or both hands on your abdomen, below the level of your umbilicus ('tummy button'). Breathe in through your nose, and as you breathe out, draw in your lower abdomen, pulling it gently away from your hands towards your back and then relax. You should be able to breathe and talk while you are doing this exercise and your lower back should stay still. You may feel the muscles working under your hand.
   - *Positions*: Start doing this exercise in the most comfortable position for you; for example, lying on your back or side with your knees bent, or sitting with your back well supported. Once you have learnt this exercise, you should be able to practise it in standing.

For peer review only

- Suggested Dosage: Start at a level that your patient can tolerate. Aiming to reach a hold 10 seconds and can repeat for a maximum of 10 times up to 3 sets.
- *Advice*: You may also feel these muscles working as you do the pelvic floor muscle exercises. Use these deep muscles by doing the hollowing throughout the day - before and during any physical activity requiring effort; for example, when lifting. If you notice any bulging of your abdominal muscles while doing any of the following 4 exercises, stop the exercise, return to abdominal hollowing and seek advice from your physiotherapist.

3) Pelvic tilt**:**

- *Exercise description*: Hollow your abdomen as described above, tighten your pelvic floor muscles, and flatten your lower back into the bed as your pelvis tilts. Breathe normally. Hold the position for 3 seconds and release gently. Repeat this up to 8-12 reps for 3 sets.
- *Position*: Progress by doing this exercise when in sitting, standing, crook lying, kneeling or lying on your side.
- Suggested Dosage: Aim for between 8-15 reps for up to 3 sets.

4) Bridge:

- *Exercise description*: Place both feet flat on the bed/floor. Hollow your abdomen as described above, tighten your pelvic floor muscles. Slowly lift your bottom off the bed to a height you are comfortable with.
- *Position*: Crook lying
- Suggested Dosage: Hold for up to 3 seconds before slowly lowering. Aim to complete 8-15 for up to 3 sets.

5) Knee bends:

- *Exercise description*: Hollow your abdomen, keep your back flat on the bed, and bend one hip and knee up as far as is comfortable. Hold up to the count of 10, and then bring the leg down so that the foot is back on the bed. Repeat with the other side.
- *Position*: crook lying
- Suggested Dosage: Hold for 10 seconds. Aim for 8-15 reps for up to 3 sets.

6) Knee rolls:

- *Exercise description*: Hollow your abdomen, and gently lower both knees to the right as far as is comfortable. Bring them back to the middle and rest for a few seconds. Hollow your abdomen again and repeat to the left.
- *Position*: crook lying
- Suggested Dosage: Aim for between 8-15 reps for up to 3 sets.

7) Head lift:

- *Exercise description*: Lie on your back with your head resting on two pillows. Hollow your abdomen and tighten the pelvic floor muscles as you gently lift your head and shoulders a little way off the pillows.
- Position: crook lying

For peer review only

- Advice: If you have neck pain, DO NOT do this exercise.
- Suggested Dosage: Hold for 3 seconds, lower and relax. Repeat between 8-15 reps for up to 3 sets.
- Progression advice: Progress by removing one pillow and ensure that you do not strain your neck muscles as you do this exercise.

8) Hip extension:
   - *Exercise description*: Participant lies prone on a flat surface with both hips in neutral and knees in full extension. Action: Hip extension against gravity
   - *Positions*: in standing with a flexed knee or in prone lying
   - *Suggested Dosage*: Aim for between 8-15 reps for a maximum of 3 sets with guidance on adding a weight or theraband as able.

9) Hip Abduction:
   - *Exercise description*: Side lying hip abduction: In a side lying position the participant abducts superficial limb to their maximum height, slowly lowers the limb back to its resting position.
   - Positions: Side lying
   - *Suggested Dosage*: Aim up to 3 sets of 8-15 reps. Position of knee bend is at therapist discretion. Perform either at 90^0 knee flexion or 0^0 extension

10) Knee Drop outs:
   - Exercise description: Hollow your abdomen, keep your back flat on the bed and feet in contact with the bed. Allow one leg at a time to drop away from your midline down towards the surface you are lying on as far as you feel able before returning back to the starting position
   - Positions: Crook lying
   - Suggested Dosage: Aim for between 8-15 reps over 3 sets.

For peer review only

Supplementary file 2: Qualitative interview topic guide

Introduction

- Thank you for taking part in this interview (Post EMaPP trial Qualitative interview or exit interview)–
- We would like to check that you have had the opportunity to read the participant information sheet provided for this part of the study
- Check that consent form is signed
- I have some questions that we would like to ask you but feel free to ask any questions at any stage.
- Recording – This meeting will be recorded if you have consented and agreement to making notes (for informal exit interview)

For peer review only

Women (intervention arm):

- Please can you tell me about your experience of being involved in the trial?
- How was your experience of wearing the shorts and advice/exercise?

Women (Control arm):

- Did the treatments meet your expectations?
- How was the experience of being randomised to the control arm?

Women both arms

- How did you find the data collection methods of the trial?

  Self-report measures,

  Twice weekly pain report measures,

  Text reminders,

  Virtual nature of the trial
- How did you find the remote based appointments?

  Were there any technical difficulties?

  If so was there anything that would have helped?

- Did you feel able to adhere to the exercise prescription from the physiotherapist?

  Would there be anything that would facilitate you adherence?

  Were there any barriers to exercise adherence?

  If so what were these?

- Did the treatments have any impact on you?

  Behaviour: activity level, function, posture, sleep

  Health: pain levels

  Psychological: mood, energy

- Final thoughts: Do you have any final points that you would like to discuss or that you feel you didn't have the opportunity to say?

Clinicians

- How did you find the process of being involved in the trial?
- How did you find process of delivering the intervention (both pelvic support shorts & exercise/advice)?
- Did the trial interventions (advice/exercise) resemble usual clinical practice?
- Is there anything else that you feel would have improved the trial?
- Final thoughts: Do you have any final points that you would like to discuss or that you feel you didn't have the opportunity to say?

Exit interviews:

What were the reason/s why you could not continue to participate with the trial?

How did you find participating in the trial?

Was there any part of the trial that if altered or improved would have led you to remain in the trial?

For peer review only

Reporting checklist for protocol of a clinical trial.

Based on the SPIRIT guidelines.

Instructions to authors

Complete this checklist by entering the page numbers from your manuscript where readers will find each of the items listed below.

Your article may not currently address all the items on the checklist. Please modify your text to include the missing information. If you are certain that an item does not apply, please write "n/a" and provide a short explanation.

Upload your completed checklist as an extra file when you submit to a journal.

In your methods section, say that you used the SPIRITreporting guidelines, and cite them as:

Chan A-W, Tetzlaff JM, Gøtzsche PC, Altman DG, Mann H, Berlin J, Dickersin K, Hróbjartsson A, Schulz KF, Parulekar WR, Krleža-Jerić K, Laupacis A, Moher D. SPIRIT 2013 Explanation and Elaboration: Guidance for protocols of clinical trials. BMJ. 2013;346:e7586

		Reporting Item	Page Number
**Administrative information**			
Title	#1	Descriptive title identifying the study design, population, interventions, and, if applicable, trial acronym	1

For peer review only

Trial registration	#2a	Trial identifier and registry name. If not yet registered, name of intended registry	2
Trial registration: data set	#2b	All items from the World Health Organization Trial Registration Data Set	Separate file
Protocol version	#3	Date and version identifier	16
Funding	#4	Sources and types of financial, material, and other support	16
Roles and responsibilities: contributorship	#5a	Names, affiliations, and roles of protocol contributors	16
Roles and responsibilities: sponsor contact information	#5b	Name and contact information for the trial sponsor	16
Roles and responsibilities: sponsor and funder	#5c	Role of study sponsor and funders, if any, in study design; collection, management, analysis, and interpretation of data; writing of the report; and the decision to submit the report for publication, including whether they will have ultimate authority over any of these activities	16
Roles and responsibilities: committees	#5d	Composition, roles, and responsibilities of the coordinating centre, steering committee, endpoint adjudication committee, data management team, and	16-17

For peer review only

		other individuals or groups overseeing the trial, if applicable (see Item 21a for data monitoring committee)	
**Introduction**			
Background and rationale	#6a	Description of research question and justification for undertaking the trial, including summary of relevant studies (published and unpublished) examining benefits and harms for each intervention	4-5
Background and rationale: choice of comparators	#6b	Explanation for choice of comparators	4-5
Objectives	#7	Specific objectives or hypotheses	5
Trial design	#8	Description of trial design including type of trial (eg, parallel group, crossover, factorial, single group), allocation ratio, and framework (eg, superiority, equivalence, non-inferiority, exploratory)	5
**Methods: Participants, interventions, and outcomes**			
Study setting	#9	Description of study settings (eg, community clinic, academic hospital) and list of countries where data will be collected. Reference to where list of study sites can be obtained	5

For peer review only

Eligibility criteria	#10	Inclusion and exclusion criteria for participants. If applicable, eligibility criteria for study centres and individuals who will perform the interventions (eg, surgeons, psychotherapists)	6
Interventions: description	#11a	Interventions for each group with sufficient detail to allow replication, including how and when they will be administered	8
Interventions: modifications	#11b	Criteria for discontinuing or modifying allocated interventions for a given trial participant (eg, drug dose change in response to harms, participant request, or improving / worsening disease)	8
Interventions: adherance	#11c	Strategies to improve adherence to intervention protocols, and any procedures for monitoring adherence (eg, drug tablet return; laboratory tests)	10-11
Interventions: concomitant care	#11d	Relevant concomitant care and interventions that are permitted or prohibited during the trial	8
Outcomes	#12	Primary, secondary, and other outcomes, including the specific measurement variable (eg, systolic blood pressure), analysis metric (eg, change from baseline, final value, time to event), method of aggregation (eg, median, proportion), and time point for each outcome. Explanation of the clinical relevance of chosen efficacy and harm outcomes is strongly recommended	9-11

For peer review only

Participant timeline	#13	Time schedule of enrolment, interventions (including any run-ins and washouts), assessments, and visits for participants. A schematic diagram is highly recommended (see Figure)	5,10
Sample size	#14	Estimated number of participants needed to achieve study objectives and how it was determined, including clinical and statistical assumptions supporting any sample size calculations	12
Recruitment	#15	Strategies for achieving adequate participant enrolment to reach target sample size	6-7
**Methods: Assignment of interventions (for controlled trials)**			
Allocation: sequence generation	#16a	Method of generating the allocation sequence (eg, computer-generated random numbers), and list of any factors for stratification. To reduce predictability of a random sequence, details of any planned restriction (eg, blocking) should be provided in a separate document that is unavailable to those who enrol participants or assign interventions	7
Allocation concealment mechanism	#16b	Mechanism of implementing the allocation sequence (eg, central telephone; sequentially numbered, opaque,	7

For peer review only

		sealed envelopes), describing any steps to conceal the sequence until interventions are assigned	
Allocation: implementation	#16c	Who will generate the allocation sequence, who will enrol participants, and who will assign participants to interventions	7
Blinding (masking)	#17a	Who will be blinded after assignment to interventions (eg, trial participants, care providers, outcome assessors, data analysts), and how	7
Blinding (masking): emergency unblinding	#17b	If blinded, circumstances under which unblinding is permissible, and procedure for revealing a participant's allocated intervention during the trial	7
**Methods: Data collection, management, and analysis**			
Data collection plan	#18a	Plans for assessment and collection of outcome, baseline, and other trial data, including any related processes to promote data quality (eg, duplicate measurements, training of assessors) and a description of study instruments (eg, questionnaires, laboratory tests) along with their reliability and validity, if known. Reference to where data collection forms can be found, if not in the protocol	9-11

For peer review only

Data collection plan: retention	#18b	Plans to promote participant retention and complete follow-up, including list of any outcome data to be collected for participants who discontinue or deviate from intervention protocols	9, 15
Data management	#19	Plans for data entry, coding, security, and storage, including any related processes to promote data quality (eg, double data entry; range checks for data values). Reference to where details of data management procedures can be found, if not in the protocol	14
Statistics: outcomes	#20a	Statistical methods for analysing primary and secondary outcomes. Reference to where other details of the statistical analysis plan can be found, if not in the protocol	14-15
Statistics: additional analyses	#20b	Methods for any additional analyses (eg, subgroup and adjusted analyses)	14-15
Statistics: analysis population and missing data	#20c	Definition of analysis population relating to protocol non-adherence (eg, as randomised analysis), and any statistical methods to handle missing data (eg, multiple imputation)	14-15
**Methods: Monitoring**			
Data monitoring: formal committee	#21a	Composition of data monitoring committee (DMC); summary of its role and reporting structure; statement of whether it is independent from the sponsor and	16

For peer review only

		competing interests; and reference to where further details about its charter can be found, if not in the protocol. Alternatively, an explanation of why a DMC is not needed	
Data monitoring: interim analysis	#21b	Description of any interim analyses and stopping guidelines, including who will have access to these interim results and make the final decision to terminate the trial	12
Harms	#22	Plans for collecting, assessing, reporting, and managing solicited and spontaneously reported adverse events and other unintended effects of trial interventions or trial conduct	11
Auditing	#23	Frequency and procedures for auditing trial conduct, if any, and whether the process will be independent from investigators and the sponsor	16-17
**Ethics and dissemination**			
Research ethics approval	#24	Plans for seeking research ethics committee / institutional review board (REC / IRB) approval	13
Protocol amendments	#25	Plans for communicating important protocol modifications (eg, changes to eligibility criteria, outcomes, analyses) to relevant parties (eg, investigators, REC / IRBs, trial participants, trial registries, journals, regulators)	13

For peer review only

Consent or assent	#26a	Who will obtain informed consent or assent from potential trial participants or authorised surrogates, and how (see Item 32)	7
Consent or assent: ancillary studies	#26b	Additional consent provisions for collection and use of participant data and biological specimens in ancillary studies, if applicable	n/a
Confidentiality	#27	How personal information about potential and enrolled participants will be collected, shared, and maintained in order to protect confidentiality before, during, and after the trial	6-7
Declaration of interests	#28	Financial and other competing interests for principal investigators for the overall trial and each study site	16
Data access	#29	Statement of who will have access to the final trial dataset, and disclosure of contractual agreements that limit such access for investigators	16
Ancillary and post trial care	#30	Provisions, if any, for ancillary and post-trial care, and for compensation to those who suffer harm from trial participation	n/a
Dissemination policy: trial results	#31a	Plans for investigators and sponsor to communicate trial results to participants, healthcare professionals, the public, and other relevant groups (eg, via publication, reporting in results databases, or other data sharing arrangements), including any publication restrictions	13

For peer review only

Dissemination policy: authorship	#31b	Authorship eligibility guidelines and any intended use of professional writers	13
Dissemination policy: reproducible research	#31c	Plans, if any, for granting public access to the full protocol, participant-level dataset, and statistical code	13
**Appendices**			
Informed consent materials	#32	Model consent form and other related documentation given to participants and authorised surrogates	n/a
Biological specimens	#33	Plans for collection, laboratory evaluation, and storage of biological specimens for genetic or molecular analysis in the current trial and for future use in ancillary studies, if applicable	n/a

None The SPIRIT Explanation and Elaboration paper is distributed under the terms of the Creative Commons Attribution License CC-BY-NC. This checklist can be completed online using https://www.goodreports.org/, a tool made by the EQUATOR Network in collaboration with Penelope.ai

For peer review only

BMJ Open

Evaluating the Management of chronic Pelvic girdle Pain following pregnancy (EMaPP): Study protocol for a randomised controlled feasibility trial to compare a customised pelvic orthosis with standard care.

Journal:	*BMJ Open*
Manuscript ID	bmjopen-2022-063767.R1
Article Type:	Protocol
Date Submitted by the Author:	24-May-2022
Complete List of Authors:	Halliday, Bradley; University of Plymouth, Faculty of Health<br>Chatfield, Sarah; University of Plymouth, Faculty of Health<br>Cameron, Lee; Aneurin Bevan Health Board<br>Hosking, Joanne; University of Plymouth, Peninsula Clinical Trials Unit<br>Shawe, Jill; Royal Cornwall Hospitals NHS Trust; University of Plymouth, Faculty of Health<br>Hawton, Annie; University of Exeter, Health Economics Group<br>Hayward, Christopher; University of Plymouth, Peninsula Clinical Trials Unit<br>Carter, Kirsty; Cornwall Partnership NHS Foundation Trust<br>Freeman, Jennifer; University of Plymouth, Faculty of Health
**Primary Subject Heading**:	Rehabilitation medicine
Secondary Subject Heading:	Obstetrics and gynaecology
Keywords:	REHABILITATION MEDICINE, PAIN MANAGEMENT, Musculoskeletal disorders < ORTHOPAEDIC & TRAUMA SURGERY

SCHOLARONE™
Manuscripts

Title: **Evaluating the Management of chronic Pelvic girdle Pain following pregnancy (EMaPP): Study protocol for a randomised controlled feasibility trial to compare a customised pelvic orthosis with standard care.**

Corresponding Author: Bradley Halliday, School of Health Professions, Faculty of Health, University of Plymouth, Derriford Road, PL6 8BH. E-mail: bradley.halliday@plymouth.ac.uk

Co-authors:

Sarah Chatfield (MSc), Faculty of Health, University of Plymouth, Plymouth, England

Lee Cameron (PhD), Aneurin Bevan University Health Board, Gwent, Wales

Joanne Hosking (PhD), Peninsula Clinical Trials Unit, Plymouth, England

Jill Shawe (PhD), Faculty of Health, University of Plymouth, Plymouth, England & Royal Cornwall Hospital NHS trust, Cornwall, England

Annie Hawton (PhD), Health Economics Group, University of Exeter Medical School, Exeter, England

Chris Hayward (BSc), Peninsula Clinical Trials Unit, Plymouth, England

Kirsty Carter BA (Hons), Cornwall Partnership NHS Foundation Trust, Cornwall, England

Jennifer Freeman (PhD), Faculty of Health, University of Plymouth, Plymouth, England

Word count: 4170

Exclusive licence

I, the Submitting Author has the right to grant and does grant on behalf of all authors of the Work (as defined in the below author licence), an exclusive licence and/or a non-exclusive licence for contributions from authors who are: i) UK Crown employees; ii) where BMJ has agreed a CC-BY licence shall apply, and/or iii) in accordance with the terms applicable for US Federal Government officers or employees acting as part of their official duties; on a worldwide, perpetual, irrevocable, royalty-free basis to BMJ Publishing Group Ltd ("BMJ") its licensees and where the relevant Journal is co-owned by BMJ to the co-owners of the Journal, to publish the Work in BMJ Open and any other BMJ products and to exploit all rights, as set out in our licence.

For peer review only - http://bmjopen.bmj.com/site/about/guidelines.xhtml

ABSTRACT

Introduction: An estimated 10% of women experience severe, chronic pelvic girdle pain post-partum. This has significant physical, psychological and socioeconomic consequences. Typically, such pain is recalcitrant to conservative management; hence the need to identify effective management strategies. Customised Dynamic Elastomeric Fabric Orthoses (DEFOs) may be an option to address this gap; designed to improve pain by providing support whilst optimising movement and function. Currently, no studies have evaluated the clinical and cost effectiveness, or acceptability of these customised orthoses in post-partum women.

Methods and analysis: EMaPP is a pragmatic, multi-centre randomised controlled feasibility trial with an embedded qualitative study and economic evaluation. Sixty participants with pregnancy related severe pelvic girdle pain > 3 months post-partum will be recruited. Participants will be randomly allocated in a 1:1 ratio (stratified by centre and presence/absence of lumbo-pelvic pain pre pregnancy) to receive either standard care (standardised information and exercise) or intervention (orthosis plus standard care). All participants will be asked to complete a battery of self-report questionnaires (including pain, function, health-related quality of life and health and social care resource use), via a web-based application at baseline, 12 weeks and 24 weeks. Pain levels and medication usage will be reported fortnightly. Feasibility and acceptability of the trial procedures will be determined in terms of recruitment and retention rates, data completion rates, and intervention adherence. Five clinicians and ten participants will be interviewed to explore their experiences of the trial procedures and receiving the intervention.

Ethics and dissemination: This study was approved by: National Research Ethics Scheme (NRES Committee Health and Care Research Wales REC (21/WM/0155) and University of Plymouth Faculty of Health Research Ethics and Integrity Committee (ref:2966). Results will be made available to participants, the funders, staff, general public and other researchers through a range of mechanisms.

Trial registration: ISRCTN67232113. Registered on 5 August 2021

Trial status: currently recruiting

Key words: Pelvic girdle pain, post-partum, Dynamic Elastomeric Fabric Orthosis, pelvic orthosis, exercise, advice, feasibility trial

STRENGTHS AND LIMITATIONS

- EMaPP is the first multi-centre randomised controlled feasibility trial assessing the feasibility and acceptability trial procedures, comparing the delivery of a novel pelvic orthosis plus standard care with standard care alone in women experiencing severe PGP post-partum
- All trial procedures and outcome measures are undertaken virtually, examining the acceptability of this approach.
- EMaPP data collection has accounted for the worst and average pain with the NRPS (in full), collected fortnightly, capturing the variability and the cyclical nature of pain experienced by participants.

For peer review only - http://bmjopen.bmj.com/site/about/guidelines.xhtml

- The trial includes an embedded qualitative element investigating acceptability of the intervention and trial procedures, with purposive sampling to capture a diversity of views from the participants and clinicians.
- A limitation is that adherence to exercise is not systematically captured

For peer review only

For peer review only - http://bmjopen.bmj.com/site/about/guidelines.xhtml

INTRODUCTION

An estimated 70% of women experience pelvic girdle pain (PGP) during pregnancy,[1] with approximately 10% of these experiencing this for longer than three months post-partum. For some this pain is severe and long-lasting, with reports of pain for more than a decade post-partum.[2-4] This has significant physical, psychological and socioeconomic consequences. Often everyday activities are affected such as moving in bed, walking, driving, breast feeding (due to discomfort and the effect of analgesia in breast milk), continence (due to associated pelvic floor dysfunction) and safely caring for the child and siblings, especially as the child becomes more mobile.[5] The emotional impacts of “living with enduring pain”, include anger and frustration and feelings of “being a burden”,[6] with related abuse of analgesics highlighted.[7, 8] Work absenteeism contributes significantly to the economic consequences.

Despite such wide-ranging impacts, symptoms can be overlooked or dismissed by health professionals believing the pain will naturally resolve.[2, 9] However, severe long-lasting pain is often recalcitrant to usual management,[10, 11] and can ultimately require invasive and expensive fluoroscopy guided injections and surgical procedures (ablation and fusion). There is an urgent need for effective strategies to address this personal and societal burden. In particular, guidance is needed for the extended post-partum phase since all current guidance relates to pain during pregnancy and up to 8 weeks post-partum.[12] In light of this, National Health Service (NHS) England has committed to improving access to postnatal physiotherapy.[13]

Pelvic orthoses are one option to manage this condition having support from European guidelines,[14] and the Royal College of Obstetricians and Gynaecologists.[15] These are externally worn devices designed to increase pelvic joint stability, musculoskeletal alignment and sensory input to optimise muscle control, pain and function.[16] There is currently a wide range of “off the shelf” pelvic orthoses available, however they have been shown to be of mixed benefit and often ineffective when pain is severe.[17] Furthermore, women report wear-time issues due to discomfort, lack of ease of use, reduced aesthetics, and impact on movement.[8] Sufficient wear time is however crucial for orthotics as benefits gained are dependent on this.

A novel customised Dynamic Elastomeric Fabric Orthosis (DEFO) for people with PGP has been designed, (DM Orthotics' Ltd, https://www.dmorthotics.com) to address these issues. This pelvic orthosis differs markedly in the design, material and compression grades to “off the shelf” pelvic orthoses. Their use has been previously assessed; during pregnancy, through a randomised controlled trial (RCT), finding that they significantly improved pain over and above the traditional rigid belt;[18] and in a replicated case series of group of eight women experiencing post-partum PGP, identifying an improvement in pain, function and quality of life.[19] Both studies indicated the pelvic DEFOs were acceptable to wear with regard to wear-time, comfort, and aesthetics. These studies together indicate a potentially effective new treatment option to managing this difficult to treat condition in the post-partum period. Further evidence is required to establish the clinical and cost effectiveness of this novel intervention in women experiencing persistent severe PGP following pregnancy. Before considering a definitive trial, the feasibility of running such a trial needs to be tested.

For peer review only - http://bmjopen.bmj.com/site/about/guidelines.xhtml

Aims and objectives

The primary aim is to assess the feasibility and acceptability of the trial procedures comparing the delivery of a novel pelvic orthosis plus standard care with standard care alone in women experiencing severe, chronic PGP post-partum.

The trial objectives are to gain operational experience and gather high quality data to inform the conduct and design of an anticipated definitive RCT and cost-effectiveness analysis, including the most appropriate primary and secondary outcome measures, the most effective recruitment methods, and to inform the sample size calculation of the future trial.

METHODS

Trial design

The EMaPP trial is an assessor blinded, pragmatic, randomised controlled feasibility trial comparing standard care (standardised advice and exercises) to standard care in addition to a customised pelvic orthosis, with an embedded qualitative study. A CONSORT study flowchart outlines the participant pathway through the trial (Figure 1). The study started in March 2021 and ends in June 2023.

Study settings

The three study sites are based in three geographical regions of England: Devon, West Yorkshire, and Buckinghamshire. The interventions are delivered in primary or secondary care subject to local practices.

The trial undertakes a remote, distance-based approach to its delivery at all stages.

- Telephone and virtual screening.
- Interventions delivered via videoconferencing by NHS physiotherapy services using locally approved software.

To prevent digital exclusion, face-to-face intervention will be delivered at a local health care establishment when required.

Eligibility criteria

The study population will comprise women with post-partum PGP, based on the eligibility criteria outlined in Table 1.

Participant recruitment, identification and screening

A multi-faceted recruitment approach will be undertaken as follows:

Nationally, social media will be used to promote the trial via a various sites including: Mumsnet, Facebook, and Twitter pages of the Pelvic, Obstetric and Gynaecological physiotherapy special interest group, National Maternity Voices, National Childbirth Trusts, and the dedicated study website (https://www.plymouth.ac.uk/research/emapp-trial).

For peer review only - http://bmjopen.bmj.com/site/about/guidelines.xhtml

For peer review only

Locally, recruitment will occur via: the Clinical Research Networks who will undertake selected GP database searches; caseloads of physiotherapists working within musculoskeletal, women's health services of the recruiting sites; posters in relevant outpatient clinics/ waiting areas of participating sites; local patient and public involvement and engagement (PPIE) groups and hospital social media accounts.

The trial will use a dedicated email address, included on all promotional material along with a QR code, to direct interested parties to further information on the trial website. The trial website contains a downloadable participant information sheet (PIS) and reply slip.

All interested participants, and those on databases or caseloads who are deemed potentially eligible, will be sent a trial information pack containing: PIS; details of the ethically approved study sites; and a reply slip to indicate their interest and confirm they feel they are eligible.

Eligibility screening process

Eligibility screening will occur via a two-stage process. On receipt of the completed reply form a telephone screen (stage 1) will be undertaken using a pre-formatted screening checklist based on the eligibility criteria. If deemed eligible, a video conference physical screening assessment will be arranged (stage 2). A screening pack will be sent out ahead of this physical screening appointment containing: the screening battery tests (schema of physical tests and instructions), Beighton self-report score, trial consent forms, orthotic measuring guide and tape measure (to capture measurements for production of the customised orthosis, should the participant be allocated to the intervention group) and digital callipers.

The physical screening requires the presence of a partner/trusted other to assist with the physical assessment and orthosis measurements, under the guidance of the remote researcher. All screen failures will be recorded.

Randomisation

Once eligibility is confirmed, the consent process completed, and screening data entered into the trial database, participants will be sent a text/email, generated by the Peninsula Clinical Trials Unit (PenCTU), requesting them to complete the baseline questionnaires. Once complete, the participant will be randomly allocated on a 1:1 ratio, using random permuted blocks, stratified by centre and presence/absence of lumbo-pelvic pain pre-pregnancy, to either the standard care or intervention group (figure 1). Screening data capture and randomisation is achieved via a web-based system created by the PenCTU.

After randomisation, a series of emails will be automatically generated. A blinded email will be sent to the research team members involved in eligibility screening. An un-blinded email will be sent to the Chief Investigator (CI), study administrator and site Principal Investigator (PI). An email/text will be sent to the participant to inform them of their group allocation.

Blinding

The trial participants and NHS treating physiotherapists are unable to be blinded due to the nature of the intervention. Every effort will be made to ensure the research team is blinded to group

For peer review only - http://bmjopen.bmj.com/site/about/guidelines.xhtml

allocation throughout the trial. The statisticians will remain blind to group allocation when conducting the primary analyses until analyses that necessitate un-blinding need to be performed, such as wear-time analysis. The research team will keep a detailed log of any inadvertent un-blinding events. Final un-blinding of the research team will occur after database lock and blinded analysis has been undertaken.

Interventions

NHS physiotherapists will provide all interventions at the two protocolised video-based sessions. At the participant's preference, face to face sessions can be undertaken in place of these remotely delivered sessions. The initial session will last a maximum of 60 minutes and the follow-up session (a maximum of 30 minutes) will be scheduled approximately 10 days later. Participants are not prevented from receiving other care. This will be captured in the health resource use questionnaire.

Standard care group (standardised information and exercise)

The standard care regime was designed in collaboration with women's health physiotherapists experienced in the treatment of persistent PGP. This was considered by them to be reflective of usual clinical practice. It comprises two components:

Standardised information:

This will be provided through a discussion centred on the publicly available booklet produced by the Pelvic, Obstetric and Gynaecological Physiotherapy special interest group "Guidance for mothers-to-be and New Mothers: Pregnancy-related Pelvic Girdle pain" (https://pogp.csp.org.uk/system/files/pogp-pgppat_3.pdf).

Standardised exercise:

The physiotherapist will teach participants a programme of up to four lumbo-pelvic/hip exercises, typical of those provided within usual physiotherapy practice. These will be selected from a protocolised set (supplementary file 1), on the basis of their initial assessment. Participants will be advised to complete the exercises on three days of the week, with each session lasting approximately 20-30 minutes. All participants will receive an exercise leaflet describing the exercises, with accompanying images. This will be posted or emailed dependent on participant preference.

Intervention group (standardised information and exercise plus DEFO)

In addition to standard care, participants allocated to the intervention group will be provided with two pairs of customised DEFOs (figure 3, DM Orthotics Ltd, https://www.dmorthotics.com). The orthosis will be worn for the first time at the initial intervention session, enabling the physiotherapist to check the fit; this will also be reviewed at the follow up session. Participants will be advised to wear the DEFO for a maximum of 12 hours per day, with wear time graduated over the course of the first five days in line with manufacturer guidance. The orthoses are delivered with an advice booklet detailing donning and doffing, wear time and care instructions, with a supplementary video hosted on the trial web page. Where alterations are required because of poor fit, in line with current practice the NHS physiotherapists will follow a standard operating procedure, making contact with the orthotic company to arrange an adjustment if required.

For peer review only - http://bmjopen.bmj.com/site/about/guidelines.xhtml

Data collection

Feasibility outcomes

The trial outcomes include feasibility and acceptability of the EMaPP trial. Quantitative and qualitative feedback will be obtained to identify the main determinants of experience and acceptance of the trial in line with the trial objectives. In particular, the following measures and operational criteria will be determined:

- Number and proportion of eligible participants from the target population.
- Willingness of participants to be randomised.
- Recruitment and retention rates of eligible participants through the trial.
- Completeness of data sets/outcome measures.
- Incidence of face to face sessions.

Clinical outcome measures

A battery of standardised, patient reported outcome measures, together with demographic and diagnostic characteristics (birth details, pain history, Beighton Score,[20, 21] and Two Point Estimation (2PE) Task,[22,23] will be collected at baseline. A selection of outcome measures will be repeated fortnightly and at 12 and 24-weeks after the first intervention session to map in more detail the trajectory of pain over the trial timeline (table 2). All patient reported outcome measures will be reported directly by the participants via the web-based app. At each time point, participants will automatically receive five text/email reminders (one per day) if the data entry has not started. All patient reported outcome measures included within this study align with the PGP core outcome set for evaluating the effectiveness of interventions in post-partum PGP.[24]

Proposed clinical outcome measures

Numerical Rating of Pain Scale (NRPS) is the proposed primary outcome measure for the definitive RCT. This 0-10 point scale is widely used,[25] quick to complete, and with a format suitable for completion through a mobile/app. There is evidence in chronic pain patients to suggest a 1-point change is clinically significant,[26] and hence a difference between groups of >1.0 at 24-week follow up will indicate a signal of efficacy. Participants will rate their pain experience over the past fortnight using the NPRS within four categories: i) worst level of day-time pelvic pain, ii) average level of day-time pelvic pain, iii) worst level of night-time pelvic pain, iv) average level of night-time pelvic pain.

The proposed secondary outcome measures are detailed in Table 2.

Resource use and costs associated with delivery of both intervention arms will be estimated. Resource use associated with the measurement, ordering, delivery and production of the orthosis, and the physiotherapy interventions, will be collected via within trial reporting, including participant level contact and non-contact time for NHS staffing input.

Intervention fidelity

For peer review only - http://bmjopen.bmj.com/site/about/guidelines.xhtml

NHS clinicians will complete a pre-formatted intervention fidelity checklist for each of the two physiotherapy sessions. Adherence to wearing the orthosis will be assessed via a temperature sensor integrated into the seams of the orthosis. The Orthotimer sensor (Rollerwerk Medical Engineering, Balingen, Germany) is embedded in a small (13mm × 9mm × 5 mm), dust and watertight unit. It will record time, date, and temperature every 15 minutes over the 24-week period with temperature precision of ± 0.1°C. DM Orthotics Ltd will activate and sew the sensor into each orthosis before dispatching to participants. Adherence to the home exercise programme is not assessed during the trial, however it is explored via the qualitative interviews.

Adverse events

Throughout the trial, all possible precautions will be taken to ensure participant safety and wellbeing. Participants will be monitored for adverse events throughout the 24-weeks. The likelihood of participants being harmed by either wearing the orthosis or undertaking the exercise programme (which reflects usual clinical practice) or any of the trial procedures is considered very low. Collection and reporting of adverse events will be captured via the web-based app and relate to those classified as an adverse reaction (including adverse device effects (ADEs)) or those which are serious. These will be collected at fortnightly intervals. Upon receipt of a reported reaction or serious adverse event, the CI will contact the local PI to discuss causality. All adverse events will be reported at the Trial Steering Committee (TSC) and Trial Management Group (TMG) meetings.

Qualitative sub-study

The aim is to explore the experiences of wearing (participant) and providing (clinicians) the pelvic orthosis and engaging in the trial procedures.

The specific objectives are to investigate acceptability of the trial methods across both arms, acceptability (comfort, wear time) of the orthosis, impact the intervention may/may not have on the participant's life, and adherence to the exercise regime.

Semi-structured telephone interviews will be undertaken, at the end of the trial, with 10 purposively sampled participants (n= 5 intervention group, n= 5 standard care group) and five NHS physiotherapists involved in delivering the interventions. A topic guide will be used (Supplementary file 2). Interviews will be digitally recorded and transcribed verbatim.

Informal telephone exit interviews will be undertaken with participants that withdraw from the trial to share their reason for withdrawing. The interviews will not be recorded or transcribed. Field notes will be taken.

End of trial definition

There are no formal stopping criteria. It will be prematurely stopped on safety grounds through a decision by the TMG and TSC.

For peer review only - http://bmjopen.bmj.com/site/about/guidelines.xhtml

Sample size

As this is a feasibility trial, a formal sample size calculation, based on power for detecting a clinically meaningful between-group difference in a primary clinical outcome, will not be undertaken. The trial aims to recruit 60 participants over a seven-month period. The sample size is pragmatic and determined to be large enough to provide robust estimates on the likely recruitment rates and follow up and estimates of the variability on the proposed primary outcome to inform a future sample size calculation. The target is set to enable determination of whether it would be practicable to recruit adequate numbers in a manner conducive to implementing a reasonably costed definitive trial. A sample size of 60 will allow overall retention rate at six-month follow-up to be estimated to within a 95% confidence interval of approximately ±13% (±10% if retention rate is 80%). Assuming a non-differential retention rate of 80%, this should provide follow-up outcome data on a minimum of 24 participants per group.

Patient and public involvement and engagement (PPIE)

Women with experience of severe chronic PGP provided input into the study design. Priority of the research question, choice of outcome measures, recruitment methods, and mode of intervention delivery were informed by discussions with women through a focus group session and ongoing discussions with a dedicated lay member of the project team. Lay input continues through membership on the TMG, and an additional independent lay member on the TSC. PPIE will play a key role in the dissemination of trial findings, contributing to and reviewing material.

Determining progression to the definitive trial

Progression to a full trial application will occur if minimum success criteria are achieved in key feasibility aims and objectives, and/or if solutions can be identified to overcome any issues. These criteria will be finalised in discussion with the TSC. A red, amber, green system will assist the decision-making progress, with Red indicating “Stop”, Amber indicating “Discuss and Modify” and Green indicating “Go”. The anticipated progression criteria are detailed in table 3.

ETHICS AND DISSEMINATION

The EMaPP trial has received approval from the: Research Ethics Committee (REC) (REC reference number: 21/WM/0155); Health Research Authority (HRA); and confirmation of capacity and capability from the Research and Development departments of the participating centres. Any amendments to the protocol will be discussed with the sponsor and submitted to HRA/REC for approval.

Dissemination will target users, clinicians and researchers. The results will inform the design of the anticipated definitive trial. It will not inform clinical decision making since clinical and cost effectiveness cannot be determined at this level. Dissemination, regardless of outcome will be via: publication of results in a peer reviewed journal, a funding proposal for a full scale effectiveness trial (if progression criteria are met), lay orientated research feedback events and a lay summary for trial participants, staff and the general public, and via conference presentations at grass roots physiotherapy events and research conferences. The study report will be accessible on the study

For peer review only - http://bmjopen.bmj.com/site/about/guidelines.xhtml

web page (https://www.plymouth.ac.uk/research/emapp-trial), as will the trial protocol and statistical analysis plan. The protocol has been written and published in line with SPIRIT guidelines.[27] Publications will follow CONSORT guidance for feasibility trials,[28] and the template for intervention description and replication (TiDIER) guidelines.[29] Authorship of the intended articles will be the study team; professional writers will not be used.

Changes post-trial registration

Initial inclusion criteria included a ceiling to pain chronicity of 2 years. This was amended to include women with pain persistence of > 2 years, following discussion with the trial steering committee, to optimise recruitment. In addition, an ethical amendment was approved for the clinical care team to contact women who had been sent a PIS but had not completed the attached reply slip; to understand the reasons behind this.

Economic evaluation

The resources required to provide the intervention will be assessed and a framework established for a future cost-effectiveness analysis alongside a definitive RCT. Data on intervention resources will be collected via within-trial reporting, including participant-level contact and non-contact time, and training for delivery staff. Participants will self-report health, social and wider care resource use, using the Resource Use Questionnaire. Participants will complete the EQ-5D-5L (the anticipated primary economic outcome measure in a full trial), and QALYs will be estimated over the follow-up period. The appropriateness of the SF-6D (based on the SF-36) for estimating QALYs will be explored. The economic evaluation methods will be developed to provide a future policy-relevant cost-effectiveness analysis of the intervention in the context of the UK NHS/Personal Social Services.

Data management and analysis

Detailed data management activities are described in a separate Data Management Plan. PenCTU staff will monitor completeness and quality of data throughout the trial. No imputation of missing data will be undertaken.

Quantitative

A detailed statistical analysis plan (SAP) will be finalised ahead of trial database locking. A CONSORT diagram will display data from screening, recruitment, consent and follow up logs and be used to generate estimates of eligibility, recruitment, consent and follow-up rates. Completion rates will be estimated for outcome measures at each time-point, including the health, social and wider care resource-use data. Recruitment and retention rates will be accompanied by 95% confidence intervals, to inform assumptions for planning the definitive trial. Adherence data (wear-time) will contribute to evaluation of intervention acceptability/feasibility and evaluated as total hours and as a percentage of total possible daytime wearing time (12 hours/day). The percentage of week's participants wore the shorts for a minimum of 42 hours a week, and the percentage of days participants wore the shorts for a minimum of 6 hours a day, will be summarised.

All outcomes will be summarised by allocated group at each follow-up with appropriate descriptive statistics. Between-group differences will be reported with 80% confidence intervals in addition to

For peer review only - http://bmjopen.bmj.com/site/about/guidelines.xhtml

95% confidence intervals, with particular focus on change in pelvic girdle day-time pain at the primary end point of 24 weeks. According to the 2008 IMMPACT consensus statement,[26] changes of one point represent minimally important decreases in pain using the NRPS of pain intensity. We will inspect the confidence interval of the between group difference to see if there is evidence of a plausible signal of efficacy. Estimates of the variability of outcome measures and the correlation between baseline and follow-up outcome measures will inform the sample size calculation for a future definitive RCT.

Qualitative

Thematic analysis will be used for the telephone interview data using NVivo 12 software for organisation and analysis (QSR International, Southport, UK, July 2021). Braun and Clarke's six-phase process will be used: (i) data familiarisation; (ii) coding; (iii) generating initial themes; (iv) reviewing themes; (v) defining and naming themes and (vi) writing up to identify patterns of meaning within the data sources. Initial themes will be refined by two researchers to maximise credibility and dependability.

Interviewees will be invited to review a draft of the analysis to ensure accurate representation of their views/experiences.

Discussion

This is the first RCT to explore the feasibility and acceptability of a novel customised pelvic orthosis in the management of chronic PGP in the post-partum period. There is a lack of evidence to support the management of this condition beyond the first eight weeks postpartum. This orthosis has already shown promise as an intervention during pregnancy,[18] and in a replicated case series study of eight women post-partum.[19] This trial aims to serve as a starting point to investigate the effectiveness of this orthosis in the management severe chronic PGP post-partum.

Some aspects of the study design are noteworthy. All trial procedures and intervention delivery are undertaken remotely; described as a virtual trial.[30, 31] This replicates current practice, mitigates the risk associated with face-to-face appointments during the COVID-19 pandemic, and ensures trial delivery in the event of a lockdown. It is also a potentially more efficient approach.[31] Because some participants may not be able to participate in a fully virtual trial, to optimise generalisability provisions have been made to provide a non-virtual format when this is preferred by the participant. Monitoring of the proportion of women who choose a non-virtual approach will further inform the future trial design.

All outcome measures collected at follow up time points (apart from adherence to wear time in the intervention group) are patient reported outcome measures (PROMs), and via a web-based app. This approach was chosen on the basis of PPIE input, the rationale being to minimise participant burden. The collection of PROMs minimises the influence of the research team on data collection. Recommendations from Mercieca-Bebber,[32] have been used to improve PROMS data collection during this trial, such as reducing free text options in questionnaires, flexible data entry, and text/email reminders to enhance data completeness. With regard to wear time adherence data, this too is collected remotely using the orthotimer temperature sensor; with the added advantage of providing detailed and objective data which is otherwise challenging to reliably collect via methods such as patient diaries.

For peer review only - http://bmjopen.bmj.com/site/about/guidelines.xhtml

The qualitative interviews are also undertaken via remote means. This decision was informed by PPIE discussions, aiming to reduce participant burden in terms of travel time and inconvenience; particularly important considerations for people in pain and looking after young children.

The self-report depression EPDS questionnaire[33] is used to assess post-natal depression. If a score of > 12 points (indicative of depression) is recorded, an email to the research team is triggered to contact the participant and their GP, thereby to ensuring duty of care is upheld. The data collected will have the added advantage of increasing understanding of the prevalence of post-natal depression in this population; currently an under-researched area.

Persistent PGP is difficult to manage with the aetiology of the condition,[34, 35] and the mechanism of action of pelvic orthotics under debate. One factor that has recently been under the spotlight within this group and may represent a possible mechanism of action for an orthotic, relates to changes in somatoperception (the perception of size and shape), with evidence pointing to an altered explicit somatoperception in both the peri-natal,[36] and post-natal period.[37] This study has included explicit (via the FreBAQ questionnaire) and implicit (measure of two-point estimation) measures of somatoperception, as recommended by Viceconti.[38] It is the first to study both types of somatoperception in parallel during an interventional trial.

Availability of data and material

The authors declare the data will be made available to the scientific community with as few restrictions as feasible, while retaining exclusive use until the publication of major outputs.

Author's Contributions:

JF is the principal investigator and together with LC, JS conceived the study. BH, JF, JS, LC, AH, CH contributed to the study design. BH, SC, LC, JH, JS, AH, KC, CH, JF are involved with the conduct of the study; BH drafted the first manuscript with all others reviewing and editing subsequent versions and the final version. JF is responsible for the overall content as guarantor.

Abbreviations: CI: Chief Investigator; CRN: Clinical Research Network; CTU: Clinical Trial Unit: DEFO: Dynamic Elastomeric Fabric Orthosis; EPDS: Edinburgh Post-natal Depression Scale: FreBAQ: Freemantle Back Awareness Questionnaires; GP: General Practitioner; ICIQ UI –SF: International Consultation Incontinence Questionnaire Urinary Incontinence - Short Form; NHS: National Health service; NRPS: Numerical Rating of Pain Scale; PGP: Pelvic Girdle Pain; PGOP: Pelvic Gynaecological and Obstetric Physiotherapists; PPIE: Patient and Public Involvement and Engagement; PGQ: Pelvic Girdle Questionnaire; PIS: Participant Information sheet; QALY: Quality Adjusted Life Year; TMG: Trial Management Group; TSC: Trial Steering Committee; TSK: Tampa Scale Kinesiophobia

Competing interests: The research team have no competing interests.

Administrative information

Protocol version: Version 3

Funding: This study is funded by the NIHR Research for Patient Benefit Programme (NIHR201930), United Kingdom. This article presents independent research. The views expressed are those of the authors and not necessarily those of the NHS, the NIHR or the Department of Health.

For peer review only - http://bmjopen.bmj.com/site/about/guidelines.xhtml

For peer review only

Name and contact information for the trial Sponsor: Royal Cornwall Hospitals NHS trust (RCHT), Research and Development Department, B18, The Knowledge Spa, Truro, Cornwall, TR1 3LJ

Role of the sponsor and funder: The sponsor RCHT assumes overall responsibility for the initiation and management of the trial. The sponsor and funder will not have direct involvement in trial design, conduct, data analysis and interpretation, manuscript writing, and dissemination of results.

Trial Steering Committee: This committee has an independent chair, an independent physiotherapist, midwife, statistician and patient representative. It meets every six months to review the progress of the trial and any serious adverse events and will report to the sponsor. A DMC is not required in this trial given the low-risk interventions.

Trial Management Group: Chaired by the Chief Investigator (Prof Freeman) and includes a representative from the sponsor, PenCTU, trial statistician, health economist and patient representative. It also has representation from co-investigators and leads for the qualitative and health economic components. The TMG will meet monthly to review trial progress and ensure appropriate management of the trial.

Supplementary file 1: Standardised exercise regime

Supplementary file 2: Topic guide for qualitative interviews

Figure Legends

Figure 1 – Trial flow chart

Figure 2 – Pelvic girdle pain screening battery

Figure 3 – Customised pelvic support shorts (DEFO)

For peer review only

For peer review only - http://bmjopen.bmj.com/site/about/guidelines.xhtml

REFERENCE LIST

1) Kovacs F, Garcia E, Royuela A, et al. Prevalence and factors associated with Low back pain and pelvic girdle pain during pregnancy: a multicentre study conducted in the Spanish national health service. *Spine* 2012;37:1516-1533 doi: 10.1097/BRS.0b013e31824dcb74
2) Wuytack F, Curtis E, Begley, C. Experiences of First-Time Mothers with Persistent Pelvic Girdle Pain after Childbirth: Descriptive Qualitative Study. *Physical Therapy 2015;*95**:** 1354-1364 doi: 10.2522/ptj.20150088
3) Bergström C, Persson M, Nergård K, et al. Prevalence and predictors of persistent pelvic girdle pain 12 years postpartum. *BMC Musculoskeletal Disorders* 2017*;*18**:**399 doi: 10.1186/s12891-017-1760-5
4) Wuytack F, Daly D, Curtis E, et al. Prognostic factors for pregnancy-related pelvic girdle pain, a systematic review. *Midwifery* 2018;66**:**70-78 doi: 10.1016/j.midw.2018.07.012
5) Mackenzie J, Murray E, Lusher J. Women's experiences of pregnancy related pelvic girdle pain: A systematic review. *Midwifery* 2018;56:102-111 doi: 10.1016/j.midw.2017.10.011
6) Persson M, Winkvist A, Dahlgren L, et al. "Struggling with daily life and enduring pain": a qualitative study of the experiences of pregnant women living with pelvic girdle pain. *BMC Pregnancy and Childbirth* 2013;13**:** 111 doi: 10.1186/1471-2393-13-111
7) Bergström C, Persson M, Mogren I. Sick leave and healthcare utilisation in women reporting pregnancy related low back pain and/or pelvic girdle pain at 14 months postpartum. *Chirop Man Therap* 2016;24:7 doi: 10.1186/s12998-016-0088-9
8) Malmqvist S, Kjaermann I, Andersen K, et al. Can a bothersome course of pelvic pain from mid-pregnancy to birth be predicted? A Norwegian prospective longitudinal SMS-Track study. *BMJ Open;* 2018:8 doi: 10.1136/bmjopen-2017-021378
9) Close C, Sinclair M, Liddle D. Women's experience of low back and/or pelvic pain (LBPP) during pregnancy. *Midwifery,* 2016; 37**:**1-8 doi: 10.1016/j.midw.2016.03.013
10) Szkwara J, Milne N, Hing W, et al. Effectiveness, Feasibility, and Acceptability of Dynamic Elastomeric Fabric Orthoses (DEFO) for Managing Pain, Functional Capacity, and Quality of Life during Prenatal and Postnatal Care: A Systematic Review. *International Journal of Environmental Research and Public Health,* 2019; 16**:** 2408 doi: 10.3390/ijerph16132408
11) Richards E, Van Kessel G, Virgara R, et al. Does antenatal physical therapy for pregnant women with low back pain or pelvic pain improve functional outcomes? A systematic review. *Acta Obstetricia et Gynecologica Scandinavica* 2012;91**:**1038-1045 doi: 10.1111/j.1600-0412.2012.01462.x
12) National Institute Clinical Excellence (NICE). Postnatal care: Clinical Guideline: 2021. https://www.nice.org.uk/guidance/ng194/resources/postnatal-care-pdf-66142082148037 Accessed: Jan 2022
13) Health Education England. The engagement of allied health professionals and psychologists in the maternity care pathway: 2019. https://www.hee.nhs.uk/ourwork/allied-health-professions/working-maximise-contribution-allied-health-professions Accessed: September 2021
14) Vleeming A, Albert H, Ostgaard, H, et al. European guidelines for the diagnosis and treatment of pelvic girdle pain. *Eur Spine J* 2008;17**:**794-819 doi: 10.1007/s00586-008-0602-4
15) Royal College of Obstetricians and Gynaecologists. https://www.nhs.uk/conditions/pregnancy-and-baby/pelvic-pain-pregnant-spd/. Accessed September 2020
16) Mens J, Pool-Goudzwaard, A. The transverse abdominal muscle is excessively active during active straight leg raising in pregnancy-related posterior pelvic girdle pain: an observational study. *BMC Musculoskelet Disord* 2017;18:372 doi: 0.1186/s12891-017-1732-9
17) Ho S, Yu W, Lao T, et al. Comfort evaluation of maternity support garments in a wear trial. *Ergonomics 2008;*51**:** 1376-1393 doi: 10.1080/00140130802116489

For peer review only - http://bmjopen.bmj.com/site/about/guidelines.xhtml

18) Cameron L, Marsden J, Watkins K, et al. Management of antenatal pelvic girdle pain study (MAPS): A double blinded randomised trial evaluating the effectiveness of two pelvic orthoses. *Int. J. Women's Health Care* 2018;3**:**1-9.
19) Cameron L. Management of Chronic Post Partum Pelvic Girdle Pain: MOPS. https://clinicaltrials.gov/ct2/show/NCT02648607
20) Cooper D, Scammell B, Batt M, et al Development and validation of self-reported line drawings of the modified Beighton score for the assessment of generalised joint hypermobility. *BMC Medical Research Methodology* 2018;18:11 doi: 10.1186/s12874-017-0464-8
21) Beighton P, Solomon L, Soskolne C. Articular mobility in an African population. *Ann Rheum Dis* 1973:32:5 413-418 doi: 10.1136/ard.32.5.413
22) Adamczyk W, Luedtke K, Saulicz O, et al. Sensory dissociation in chronic low back pain: Two case reports. *Physiotherapy Theory and Practice***,** 2018;34:8 1-9 doi: 10.1080/09593985.2017.1423431
23) Adamczyk W, Sługocka A, Mehlich K, et al. Preliminary Validation of a Two-Point Estimation Task for the Measurement of Sensory Dissociation in Patients with Chronic Low Back Pain. *Pain Medicine* 2019;20**:**2472-2478 doi: 10.1093/pm/pny220
24) Remus A, Smith V, Gutke A, et al. A core outcome set for research and clinical practice in women with pelvic girdle pain: PGP-COS. *PLOS ONE,* 2021;16:2 doi: 10.1371/journal.pone.0247466
25) Childs J, Piva S, Fritz J. Responsiveness of the numeric pain rating scale in patients with low back pain. *Spine 2005 (Phila Pa 1976);*30**:**1331-4 doi: 10.1097/01.brs.0000164099.92112.29
26) Dworkin R, Turk D, Wyrwich K, et al. Interpreting the clinical importance of treatment outcomes in chronic pain clinical trials: IMMPACT recommendations. *Journal of Pain,* 2008 9**,** 105-21 doi: 10.1016/j.jpain.2007.09.005
27) Chan A, Tetzlaff J, Altman D, et al. SPIRIT 2013 statement: defining standard protocol items for clinical trials. *Ann Intern* Med 2013;38:(6) 506–14 doi: 10.7326/0003-4819-158-3-201302050-00583
28) Eldridge S, Chan C, Campbell M, et al. CONSORT 2010 statement: extension to randomised pilot and feasibility trials. *BMJ* 2016; 355;i5239 doi: 10.1136/bmj.i5239
29) Hoffmann T, Glasziou P, Boutron I, et al. Better reporting of interventions: template for intervention description and replication (TIDieR) checklist and guide. *BMJ* 2014 348**,** g1687-g1687 doi: 10.1136/bmj.g1687
30) National Academies of Sciences, E., Medicine, Health, Medicine, D., Board on health sciences, P., Forum on drug discovery, d. & translation 2019. The National Academies Collection: Reports funded by National Institutes of Health. *In:* Shore, C., Khandekar, E. & Alper, J. (eds.) *Virtual Clinical Trials: Challenges and Opportunities: Proceedings of a Workshop.* Washington (DC): National Academies Press
31) Dorsey E, Wagner J, Bull M, et al. Feasibility of Virtual Research Visits in Fox Trial Finder. *J Parkinsons Dis* 2015;5**:**505-15 doi:10.3233/JPD-150549
32) Mercieca-bebber R, Palmer M, Brundage M, et al. Design, implementation and reporting strategies to reduce the instance and impact of missing patient-reported outcome (PRO) data: a systematic review. *BMJ Open* 2016: 6, e010938 doi: 10.1136/bmjopen-2015-010938
33) Cox J, Holden J, Sagovsky R. Detection of Postnatal Depression. *British Journal of Psychiatry* 1987;150**:**782-786 doi: 10.1192/bjp.150.6.782
34) Meijer O, Hu H, Wu W, et al. The pelvic girdle pain deadlock: 1. Would 'deconstruction' help? *Musculoskeletal Science &* Practice 2020;48**:** 102169 doi: 10.1016/j.msksp.2020.102169
35) Meijer O, Barbe M, Prins M, et al. The Pelvic Girdle Pain deadlock: 2. Topics that, so far, have remained out of focus. *Musculoskeletal Science &* Practice 2020;48**:** 102166 doi: 10.1016/j.msksp.2020.102166

For peer review only - http://bmjopen.bmj.com/site/about/guidelines.xhtml

36) Wand B, Elliott R, Sawyer A, et al. Disrupted body-image and pregnancy-related lumbopelvic pain. A preliminary investigation. *Musculoskeletal Science and Practice* 2017;30:49-55 doi: 10.1016/j.msksp.2017.05.003
37) Beales D, Lutz A, Thompson J, et al. Disturbed body perception, reduced sleep, and kinesiophobia in subjects with pregnancy-related persistent lumbopelvic pain and moderate levels of disability: An exploratory study. *Manual Therapy* 2016;21:69-75 doi: 10.1016/j.math.2015.04.016
38) Viceconti A, Camerone E, Luzzi D, et al. Explicit and Implicit Own's Body and Space Perception in Painful Musculoskeletal Disorders and Rheumatic Diseases: A Systematic Scoping Review. *Frontiers in Human Neuroscience* 2020;14:83 doi: 10.3389/fnhum.2020.00083
39) Fagevik Olsén M, Körnung P, Kallin S, et al. Validation of self-administered tests for screening for chronic pregnancy-related pelvic girdle pain. *BMC Musculoskeletal Disorders,* 2021;22: 237 doi: 10.1186/s12891-021-04103-0
40) Stuge B, Garratt A, Krogstad Jenssen H, et al. The Pelvic Girdle Questionnaire: A Condition-Specific Instrument for Assessing Activity Limitations and Symptoms in People with Pelvic Girdle Pain. *Physical Therapy* 2011;91:1096-1108 doi: 10.2522/ptj.20100357
41) Wuytack F, O'Donovan M. Outcomes and outcomes measurements used in intervention studies of pelvic girdle pain and lumbopelvic pain: a systematic review. *Chirop Man Therap* 2019;27:62 doi: 10.1186/s12998-019-0279-2
42) Whitehead S, Ali S. Health outcomes in economic evaluation: the QALY and utilities. *British medical bulletin* 2010;*96*:5–21 doi: https://doi.org/10.1093/bmb/ldq033
43) Avery K, Donovan J, Peters T, et al. ICIQ: A brief and robust measure for evaluating the symptoms and impact of urinary incontinence. *Neurourology and Urodynamics* 2004;23: 322-330 doi: 10.1002/nau.20041
44) Miller R, Kori S, Todd D. The Tampa Scale: a Measure of Kinesiophobia. *The Clinical Journal of Pain* 1991;7:51.

For peer review only

Table 1 EMaPP study inclusion and exclusion criteria	
**Inclusion criteria**	**Exclusion criteria**
Aged ≥ 18 years	Known allergy to Lycra
Able and willing to provide informed consent	Currently pregnant
Self-report persistent PGP (for a minimum of three months post-partum)	Currently wearing a catheter
Self-reported severe PGP (causing walking or stair climbing to be bothersome)	Self-reported history of pathologies causative of lumbo-pelvic pain (e.g. infection, trauma, cancer)
Diagnosis of PGP in line with European guidelines; defined as pain between the posterior iliac crest and inferior gluteal fold, particularly in the sacroiliac joint vicinity, that may radiate to the posterior thigh and occur in conjunction with or separately in the symphysis pubis,[14] captured using the pain referral map.	Participating in concurrent interventional research which may over-burden the patient or confound data collection
Scoring positively with at least one anterior PGP test and two tests for posterior PGP or two positive tests for posterior PGP.[39] [Figure 2]	Participants unable to understand verbal and/or written English adequate to complete the trial procedures/self-report questionnaires. Assistance from a friend/family member for translation purposes is acceptable.
The PGP must have started or been aggravated during pregnancy, as determined by self-report.	

Table 2: Assessment schedule

**Assessments**	Baseline	Post-initial appointment		
		Fortnightly	12 weeks	24 weeks
Demographics, birth details, pain history	x			
Beighton Score[20, 21] to determine joint hypermobility	x			
Two-point estimation task[22, 23] to determine implicit somatoperception	x			x
Pain medication use	x	x	x	x
Numerical Pain Rating Scale [25, 26]	x	x	x	x

For peer review only - http://bmjopen.bmj.com/site/about/guidelines.xhtml

Health and Social Care Resource use Questionnaire to collect data on health, social and wider care resource use[18]	x		x	x
Edinburgh Post-natal Depression Scale [33] to assess post-natal depression (cut-off ≥ 12 points)	x		x	x
Freemantle Back Awareness Questionnaire to assess explicit body perception of the lumbo-pelvic region[36,37]	x		x	x
Pelvic Girdle Questionnaire [40] to assess function and symptoms	x		x	x
Short-form 36 [41] to assess health-related quality of life and calculate quality adjusted life-years (QALYS)	x		x	x
EQ-5D-5L to assess health-related quality of life and calculate QALYS [42]	x		x	x
International Consultation on Incontinence Questionnaire, Urinary Incontinence - Short form to assess level and impact of symptoms of incontinence [43]	x		x	x
Tampa Scale Kinesiophobia to assess fear of movement/kinesiophobia[44]	x		x	x
Wear time adherence (Orthotimer)				x
Qualitative interviews				x

Table 3: Progression criteria

Criteria	RAG rating
60 participants recruited within a 7-month recruitment window	Red: <60%<br>Amber: 60-80%<br>Green: > 80%
Percentage of participants randomised to intervention group non-compliant in wearing the shorts (non-compliance – wearing the shorts for less than 6 hours/day or total of 42 hours/week)	Red: > 70%<br>Amber: 50-70%<br>Green: <50%
Percentage of participants completing primary outcome measure (NRPS) at 24-week follow up	Red: < 60%<br>Amber: 60-80%<br>Green: > 80%
* Percentage of participants completing secondary outcome measures at 24 week follow-up in (in the following order of priority (EQ-5D-5L, SF-36, PGQ, ICIQ)	Red < 60%,<br>Amber 60-80%,<br>Green > 80%
Evidence to suggest efficacy, i.e. that the support shorts hold promise as an effective intervention, demonstrated by an 80% confidence interval that indicates plausibility of the between group difference in the primary outcome measure being ≥ 1 point, on the NPRS.	N/A

For peer review only

For peer review only - http://bmjopen.bmj.com/site/about/guidelines.xhtml

Total resource estimated to conduct the definitive trial within levels likely to attract funding.	N/A

RAG rating = Red Amber Green Rating

Table 3: Progression criteria. *This progression criteria relates to the selection of outcome measures to be used in a definitive trial. It does not influence the decision as to whether or not there should be progression to a definitive trial.

For peer review only

For peer review only - http://bmjopen.bmj.com/site/about/guidelines.xhtml

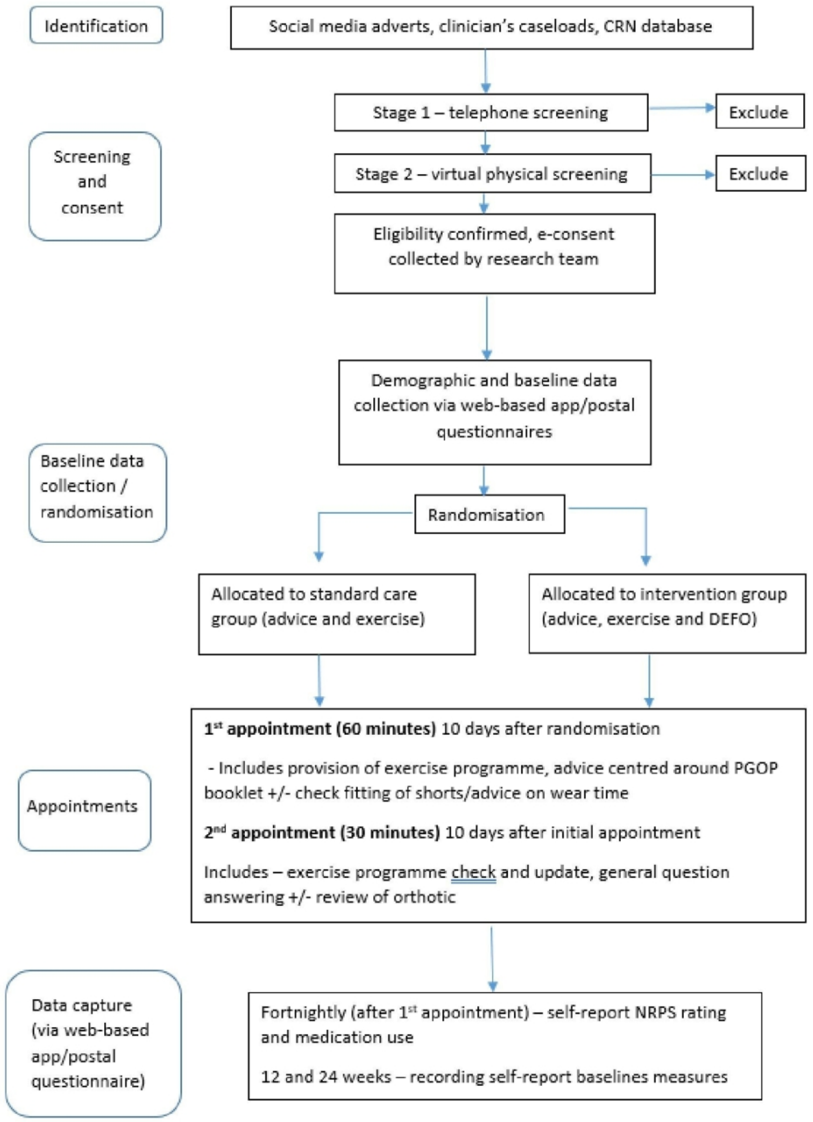

Trial flow chart

159x200mm (300 x 300 DPI)

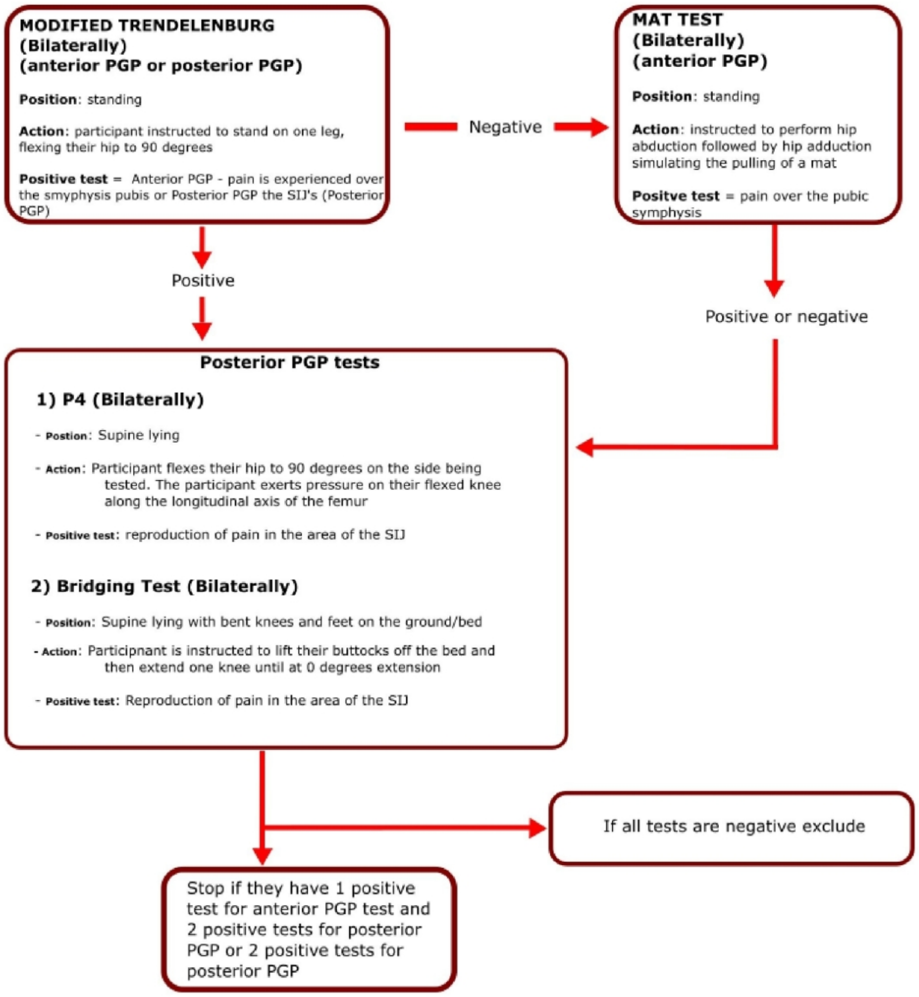

Pelvic girdle pain screening battery

159x200mm (300 x 300 DPI)

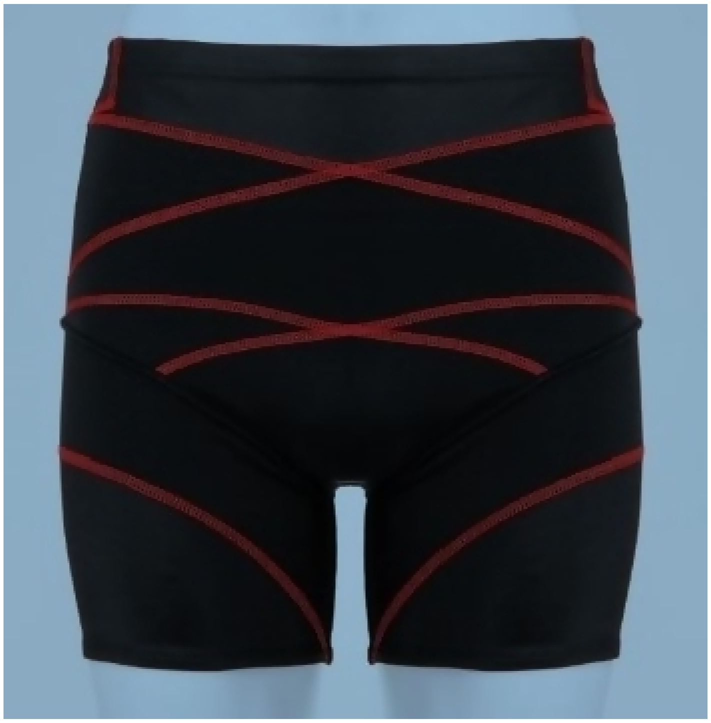

Customised pelvic support shorts (DEFO)

29x30mm (300 x 300 DPI)

Supplementary file 1: Standardised Exercise regime

Reference: Exercises adapted from "Exercise and Advice after pregnancy" PGOP booklet. https://pogp.csp.org.uk/system/files/publication_files/POGP-E%26AA5%28UL%29.pdf

Location: completed at home independently.

Frequency: Aim x 3 per week

Dosage: Aim for up 20 - 30 min's per session. Participants may need to break this into 2 parts.

Repetitions: Exercises described have suggested sets and repetitions. However, this should be guided by treating clinician and the women's capacity to exercise.

Quantity: Choose a maximum of 4 exercises

Advice to be provided to all women: If any of the exercises cause pain then reduce the amount of repetitions that you are performing. If this continues on more than two occasions, then cease the exercise and discuss with your clinician at your follow up appointment.

Permitted exercises are described below:

1) Pelvic floor exercises
    - *Exercise description*: Imagine that you are trying to stop yourself passing urine or wind: 'squeeze and lift' your pelvic floor muscles, closing and drawing up the 3 passages. You may not feel that much is happening at first but keep trying. Hold the squeeze for a few seconds, and then relax for a few seconds. Do not hold your breath. You may find that abdominal hollowing takes place at the same time.
    - *Positions* – consider the use of different position to exercise in (e.g. standing, sitting and lying),
    - *Speed of contraction/Dosage*:
        - *For endurance:* Start gently and rhythmically. Gradually increase the hold time and the number that you do until you can hold the squeeze for up to 10 seconds and repeat up to 15 times in a set (up to 3 sets)
        - *For speed*: It is also important that the pelvic floor muscles are able to react quickly to stop you leaking when you cough, sneeze, laugh or shout. Tighten them as quickly and strongly as you can, and then relax. Do this between 8-15 times a set with up to 3 sets.
    - *Advice*: Always tighten your pelvic floor muscles before and during any activity requiring physical effort; for example, when lifting, coughing or sneezing

2) Abdominal hollowing exercise:
    - *Exercise description*: Place one or both hands on your abdomen, below the level of your umbilicus ('tummy button'). Breathe in through your nose, and as you breathe out, draw in your lower abdomen, pulling it gently away from your hands towards your back and then relax. You should be able to breathe and talk while you are doing this exercise and your lower back should stay still. You may feel the muscles working under your hand.
    - *Positions*: Start doing this exercise in the most comfortable position for you; for example, lying on your back or side with your knees bent, or sitting with your back well supported. Once you have learnt this exercise, you should be able to practise it in standing.

For peer review only

- Suggested Dosage: Start at a level that your patient can tolerate. Aiming to reach a hold 10 seconds and can repeat for a maximum of 10 times up to 3 sets.
- *Advice*: You may also feel these muscles working as you do the pelvic floor muscle exercises. Use these deep muscles by doing the hollowing throughout the day - before and during any physical activity requiring effort; for example, when lifting. If you notice any bulging of your abdominal muscles while doing any of the following 4 exercises, stop the exercise, return to abdominal hollowing and seek advice from your physiotherapist.

3) Pelvic tilt**:**

- *Exercise description*: Hollow your abdomen as described above, tighten your pelvic floor muscles, and flatten your lower back into the bed as your pelvis tilts. Breathe normally. Hold the position for 3 seconds and release gently. Repeat this up to 8-12 reps for 3 sets.
- *Position*: Progress by doing this exercise when in sitting, standing, crook lying, kneeling or lying on your side.
- Suggested Dosage: Aim for between 8-15 reps for up to 3 sets.

4) Bridge:

- *Exercise description*: Place both feet flat on the bed/floor. Hollow your abdomen as described above, tighten your pelvic floor muscles. Slowly lift your bottom off the bed to a height you are comfortable with.
- *Position*: Crook lying
- Suggested Dosage: Hold for up to 3 seconds before slowly lowering. Aim to complete 8-15 for up to 3 sets.

5) Knee bends:

- *Exercise description*: Hollow your abdomen, keep your back flat on the bed, and bend one hip and knee up as far as is comfortable. Hold up to the count of 10, and then bring the leg down so that the foot is back on the bed. Repeat with the other side.
- *Position*: crook lying
- Suggested Dosage: Hold for 10 seconds. Aim for 8-15 reps for up to 3 sets.

6) Knee rolls:

- *Exercise description*: Hollow your abdomen, and gently lower both knees to the right as far as is comfortable. Bring them back to the middle and rest for a few seconds. Hollow your abdomen again and repeat to the left.
- *Position*: crook lying
- Suggested Dosage: Aim for between 8-15 reps for up to 3 sets.

7) Head lift:

- *Exercise description*: Lie on your back with your head resting on two pillows. Hollow your abdomen and tighten the pelvic floor muscles as you gently lift your head and shoulders a little way off the pillows.
- Position: crook lying

For peer review only

- Advice: If you have neck pain, DO NOT do this exercise.
- Suggested Dosage: Hold for 3 seconds, lower and relax. Repeat between 8-15 reps for up to 3 sets.
- Progression advice: Progress by removing one pillow and ensure that you do not strain your neck muscles as you do this exercise.

8) Hip extension:
   - *Exercise description*: Participant lies prone on a flat surface with both hips in neutral and knees in full extension. Action: Hip extension against gravity
   - *Positions*: in standing with a flexed knee or in prone lying
   - *Suggested Dosage*: Aim for between 8-15 reps for a maximum of 3 sets with guidance on adding a weight or theraband as able.

9) Hip Abduction:
   - *Exercise description*: Side lying hip abduction: In a side lying position the participant abducts superficial limb to their maximum height, slowly lowers the limb back to its resting position.
   - Positions: Side lying
   - *Suggested Dosage*: Aim up to 3 sets of 8-15 reps. Position of knee bend is at therapist discretion. Perform either at 90^0 knee flexion or 0^0 extension

10) Knee Drop outs:
   - Exercise description: Hollow your abdomen, keep your back flat on the bed and feet in contact with the bed. Allow one leg at a time to drop away from your midline down towards the surface you are lying on as far as you feel able before returning back to the starting position
   - Positions: Crook lying
   - Suggested Dosage: Aim for between 8-15 reps over 3 sets.

For peer review only

Supplementary file 2: Qualitative interview topic guide

Introduction

- Thank you for taking part in this interview (Post EMaPP trial Qualitative interview or exit interview)–
- We would like to check that you have had the opportunity to read the participant information sheet provided for this part of the study
- Check that consent form is signed
- I have some questions that we would like to ask you but feel free to ask any questions at any stage.
- Recording – This meeting will be recorded if you have consented and agreement to making notes (for informal exit interview)

Women (intervention arm):

- Please can you tell me about your experience of being involved in the trial?
- How was your experience of wearing the shorts and advice/exercise?

Women (Control arm):

- Did the treatments meet your expectations?
- How was the experience of being randomised to the control arm?

Women both arms

- How did you find the data collection methods of the trial?

  Self-report measures,

  Twice weekly pain report measures,

  Text reminders,

  Virtual nature of the trial
- How did you find the remote based appointments?

  Were there any technical difficulties?

  If so was there anything that would have helped?

For peer review only

- Did you feel able to adhere to the exercise prescription from the physiotherapist?

  Would there be anything that would facilitate you adherence?

  Were there any barriers to exercise adherence?

  If so what were these?

- Did the treatments have any impact on you?

  Behaviour: activity level, function, posture, sleep

  Health: pain levels

  Psychological: mood, energy

- Final thoughts: Do you have any final points that you would like to discuss or that you feel you didn't have the opportunity to say?

Clinicians

- How did you find the process of being involved in the trial?
- How did you find process of delivering the intervention (both pelvic support shorts & exercise/advice)?
- Did the trial interventions (advice/exercise) resemble usual clinical practice?
- Is there anything else that you feel would have improved the trial?
- Final thoughts: Do you have any final points that you would like to discuss or that you feel you didn't have the opportunity to say?

Exit interviews:

What were the reason/s why you could not continue to participate with the trial?

How did you find participating in the trial?

Was there any part of the trial that if altered or improved would have led you to remain in the trial?

For peer review only

Reporting checklist for protocol of a clinical trial.

Based on the SPIRIT guidelines.

Instructions to authors

Complete this checklist by entering the page numbers from your manuscript where readers will find each of the items listed below.

Your article may not currently address all the items on the checklist. Please modify your text to include the missing information. If you are certain that an item does not apply, please write "n/a" and provide a short explanation.

Upload your completed checklist as an extra file when you submit to a journal.

In your methods section, say that you used the SPIRITreporting guidelines, and cite them as:

Chan A-W, Tetzlaff JM, Gøtzsche PC, Altman DG, Mann H, Berlin J, Dickersin K, Hróbjartsson A, Schulz KF, Parulekar WR, Krleža-Jerić K, Laupacis A, Moher D. SPIRIT 2013 Explanation and Elaboration: Guidance for protocols of clinical trials. BMJ. 2013;346:e7586

For peer review only

		Reporting Item	Page Number
**Administrative information**			
Title	#1	Descriptive title identifying the study design, population, interventions, and, if applicable, trial acronym	1

Trial registration	#2a	Trial identifier and registry name. If not yet registered, name of intended registry	2
Trial registration: data set	#2b	All items from the World Health Organization Trial Registration Data Set	Separate file
Protocol version	#3	Date and version identifier	16
Funding	#4	Sources and types of financial, material, and other support	16
Roles and responsibilities: contributorship	#5a	Names, affiliations, and roles of protocol contributors	16
Roles and responsibilities: sponsor contact information	#5b	Name and contact information for the trial sponsor	16
Roles and responsibilities: sponsor and funder	#5c	Role of study sponsor and funders, if any, in study design; collection, management, analysis, and interpretation of data; writing of the report; and the decision to submit the report for publication, including whether they will have ultimate authority over any of these activities	16
Roles and responsibilities: committees	#5d	Composition, roles, and responsibilities of the coordinating centre, steering committee, endpoint adjudication committee, data management team, and	16-17

For peer review only

		other individuals or groups overseeing the trial, if applicable (see Item 21a for data monitoring committee)	
**Introduction**			
Background and rationale	#6a	Description of research question and justification for undertaking the trial, including summary of relevant studies (published and unpublished) examining benefits and harms for each intervention	4-5
Background and rationale: choice of comparators	#6b	Explanation for choice of comparators	4-5
Objectives	#7	Specific objectives or hypotheses	5
Trial design	#8	Description of trial design including type of trial (eg, parallel group, crossover, factorial, single group), allocation ratio, and framework (eg, superiority, equivalence, non-inferiority, exploratory)	5
**Methods: Participants, interventions, and outcomes**			
Study setting	#9	Description of study settings (eg, community clinic, academic hospital) and list of countries where data will be collected. Reference to where list of study sites can be obtained	5

For peer review only

Eligibility criteria	#10	Inclusion and exclusion criteria for participants. If applicable, eligibility criteria for study centres and individuals who will perform the interventions (eg, surgeons, psychotherapists)	5-6
Interventions: description	#11a	Interventions for each group with sufficient detail to allow replication, including how and when they will be administered	8
Interventions: modifications	#11b	Criteria for discontinuing or modifying allocated interventions for a given trial participant (eg, drug dose change in response to harms, participant request, or improving / worsening disease)	8
Interventions: adherance	#11c	Strategies to improve adherence to intervention protocols, and any procedures for monitoring adherence (eg, drug tablet return; laboratory tests)	10-11
Interventions: concomitant care	#11d	Relevant concomitant care and interventions that are permitted or prohibited during the trial	8
Outcomes	#12	Primary, secondary, and other outcomes, including the specific measurement variable (eg, systolic blood pressure), analysis metric (eg, change from baseline, final value, time to event), method of aggregation (eg, median, proportion), and time point for each outcome. Explanation of the clinical relevance of chosen efficacy and harm outcomes is strongly recommended	9-11

For peer review only

Participant timeline	#13	Time schedule of enrolment, interventions (including any run-ins and washouts), assessments, and visits for participants. A schematic diagram is highly recommended (see Figure)	5 (figure 1) ,10
Sample size	#14	Estimated number of participants needed to achieve study objectives and how it was determined, including clinical and statistical assumptions supporting any sample size calculations	12
Recruitment	#15	Strategies for achieving adequate participant enrolment to reach target sample size	6-7
**Methods: Assignment of interventions (for controlled trials)**			
Allocation: sequence generation	#16a	Method of generating the allocation sequence (eg, computer-generated random numbers), and list of any factors for stratification. To reduce predictability of a random sequence, details of any planned restriction (eg, blocking) should be provided in a separate document that is unavailable to those who enrol participants or assign interventions	7
Allocation concealment mechanism	#16b	Mechanism of implementing the allocation sequence (eg, central telephone; sequentially numbered, opaque,	7

For peer review only

		sealed envelopes), describing any steps to conceal the sequence until interventions are assigned	
Allocation: implementation	#16c	Who will generate the allocation sequence, who will enrol participants, and who will assign participants to interventions	7
Blinding (masking)	#17a	Who will be blinded after assignment to interventions (eg, trial participants, care providers, outcome assessors, data analysts), and how	7
Blinding (masking): emergency unblinding	#17b	If blinded, circumstances under which unblinding is permissible, and procedure for revealing a participant's allocated intervention during the trial	7
**Methods: Data collection, management, and analysis**			
Data collection plan	#18a	Plans for assessment and collection of outcome, baseline, and other trial data, including any related processes to promote data quality (eg, duplicate measurements, training of assessors) and a description of study instruments (eg, questionnaires, laboratory tests) along with their reliability and validity, if known. Reference to where data collection forms can be found, if not in the protocol	9-11

For peer review only

Data collection plan: retention	#18b	Plans to promote participant retention and complete follow-up, including list of any outcome data to be collected for participants who discontinue or deviate from intervention protocols	9, 15
Data management	#19	Plans for data entry, coding, security, and storage, including any related processes to promote data quality (eg, double data entry; range checks for data values). Reference to where details of data management procedures can be found, if not in the protocol	14
Statistics: outcomes	#20a	Statistical methods for analysing primary and secondary outcomes. Reference to where other details of the statistical analysis plan can be found, if not in the protocol	14-15
Statistics: additional analyses	#20b	Methods for any additional analyses (eg, subgroup and adjusted analyses)	14-15
Statistics: analysis population and missing data	#20c	Definition of analysis population relating to protocol non-adherence (eg, as randomised analysis), and any statistical methods to handle missing data (eg, multiple imputation)	14-15

Methods: Monitoring

Data monitoring: formal committee	#21a	Composition of data monitoring committee (DMC); summary of its role and reporting structure; statement of whether it is independent from the sponsor and	16

For peer review only

		competing interests; and reference to where further details about its charter can be found, if not in the protocol. Alternatively, an explanation of why a DMC is not needed	
Data monitoring: interim analysis	#21b	Description of any interim analyses and stopping guidelines, including who will have access to these interim results and make the final decision to terminate the trial	12
Harms	#22	Plans for collecting, assessing, reporting, and managing solicited and spontaneously reported adverse events and other unintended effects of trial interventions or trial conduct	11
Auditing	#23	Frequency and procedures for auditing trial conduct, if any, and whether the process will be independent from investigators and the sponsor	16-17
**Ethics and dissemination**			
Research ethics approval	#24	Plans for seeking research ethics committee / institutional review board (REC / IRB) approval	13
Protocol amendments	#25	Plans for communicating important protocol modifications (eg, changes to eligibility criteria, outcomes, analyses) to relevant parties (eg, investigators, REC / IRBs, trial participants, trial registries, journals, regulators)	13

For peer review only

Consent or assent	#26a	Who will obtain informed consent or assent from potential trial participants or authorised surrogates, and how (see Item 32)	7
Consent or assent: ancillary studies	#26b	Additional consent provisions for collection and use of participant data and biological specimens in ancillary studies, if applicable	n/a
Confidentiality	#27	How personal information about potential and enrolled participants will be collected, shared, and maintained in order to protect confidentiality before, during, and after the trial	6-7
Declaration of interests	#28	Financial and other competing interests for principal investigators for the overall trial and each study site	16
Data access	#29	Statement of who will have access to the final trial dataset, and disclosure of contractual agreements that limit such access for investigators	16
Ancillary and post trial care	#30	Provisions, if any, for ancillary and post-trial care, and for compensation to those who suffer harm from trial participation	n/a
Dissemination policy: trial results	#31a	Plans for investigators and sponsor to communicate trial results to participants, healthcare professionals, the public, and other relevant groups (eg, via publication, reporting in results databases, or other data sharing arrangements), including any publication restrictions	13

For peer review only

Dissemination policy: authorship	#31b	Authorship eligibility guidelines and any intended use of professional writers	13
Dissemination policy: reproducible research	#31c	Plans, if any, for granting public access to the full protocol, participant-level dataset, and statistical code	13

Appendices

Informed consent materials	#32	Model consent form and other related documentation given to participants and authorised surrogates	Attached as sperate file
Biological specimens	#33	Plans for collection, laboratory evaluation, and storage of biological specimens for genetic or molecular analysis in the current trial and for future use in ancillary studies, if applicable	n/a

None The SPIRIT Explanation and Elaboration paper is distributed under the terms of the Creative Commons Attribution License CC-BY-NC. This checklist can be completed online using https://www.goodreports.org/, a tool made by the EQUATOR Network in collaboration with Penelope.ai

For peer review only
