# Supplementary material for: Evaluating the Management of chronic Pelvic girdle Pain following pregnancy (EMaPP): study protocol for a randomised controlled feasibility trial to compare a customised pelvic orthosis with standard care
Source: BMJ Open. 2022 Aug 4;12(8):e063767. doi: 10.1136/bmjopen-2022-063767 (PMC9361753; doi:10.1136/bmjopen-2022-063767)
Supplement: Reviewer comments [file bmjopen-2022-063767.reviewer_comments.pdf]

PEER REVIEW HISTORY

BMJ Open publishes all reviews undertaken for accepted manuscripts. Reviewers are asked to complete a checklist review form (**http://bmjopen.bmj.com/site/about/resources/checklist.pdf**) and are provided with free text boxes to elaborate on their assessment. These free text comments are reproduced below.

ARTICLE DETAILS

**TITLE (PROVISIONAL)**	Evaluating the Management of chronic Pelvic girdle Pain following pregnancy (EMaPP): Study protocol for a randomised controlled feasibility trial to compare a customised pelvic orthosis with standard care.
**AUTHORS**	Halliday, Bradley; Chatfield, Sarah; Cameron, Lee; Hosking, Joanne; Shawe, Jill; Hawton, Annie; Hayward, Christopher; Carter, Kirsty; Freeman, Jennifer

This article was not externally reviewed at BMJ Open. Protocols that have been independently assessed prior to submission to BMJ Open are usually fast-tracked to publication on the grounds that further substantial changes will not be possible. This independent assessment will usually be external, independent review for both a substantial grant award from a non-commercial or government funder and ethics approval.
